# Repercussions of the COVID-19 pandemic on health professionals in the state of Rio de Janeiro / Brazil

**DOI:** 10.1371/journal.pone.0261814

**Published:** 2022-01-21

**Authors:** Karla Gonçalves Camacho, Saint Clair dos Santos Gomes Junior, Adriana Teixeira Reis, Maria de Fátima Junqueira-Marinho, Luiz Carlos Moraes França, Dimitri Marques Abramov, Zina Maria Almeida de Azevedo, Maria Elisabeth Lopes Moreira, Zilton Farias Meira de Vasconcelos, Margarida dos Santos Salú, Milene Lucio da Silva, Barbara da Silveira Madeira de Castro, Juliana Martins Rodrigues, Cláudia Dayube Pereira, Jairo Werner Junior, Rossy Moreira Bastos Junior, Daniella Mancino da Luz Caixeta, Daniella Campelo Batalha Cox Moore

**Affiliations:** 1 Department of Pediatrics, National Institute of Health for Women, Children and Adolescents Fernandes Figueira (IFF / Fiocruz), City of Rio de Janeiro, Rio de Janeiro, Brazil; 2 Department of Perinatonology, State University of Rio de Janeiro, City of Rio de Janeiro, Rio de Janeiro, Brazil; 3 Clinical Research Department, National Institute of Health for Women, Children and Adolescents Fernandes Figueira (IFF / Fiocruz), City of Rio de Janeiro, Rio de Janeiro, Brazil; 4 Department of Education, National Institute of Health for Women, Children and Adolescents Fernandes Figueira (IFF / Fiocruz), City of Rio de Janeiro, Rio de Janeiro, Brazil; 5 Department of Pediatrics, University of Grande Rio, UNIGRANRIO, City of Rio de Janeiro, Rio de Janeiro, Brazil; 6 High Complexity Laboratory, National Institute of Health for Women, Children and Adolescents Fernandes Figueira (IFF / Fiocruz), City of Rio de Janeiro, Rio de Janeiro, Brazil; 7 Department of Pediatrics, Ismélia da Silveira Children’s Hospital, City of Rio de Janeiro, Rio de Janeiro, Brazil; 8 Department of Psychology, National Institute of Health for Women, Children and Adolescents Fernandes Figueira (IFF / Fiocruz), City of Rio de Janeiro, Rio de Janeiro, Brazil; 9 Faculty of Medicine, Federal Fluminense University (UFF), City of Niteroi, Rio de Janeiro, Brazil; 10 Universidade Iguaçu, UNIG, and Macaé City Hall RJ, City of Macaé, Rio de Janeiro, RJ–Brazil; 11 Federal Fluminense University (UFF), City of Rio de Janeiro, Rio de Janeiro, Brazil; Sunway University, MALAYSIA

## Abstract

Brazil has been severely affected by the COVID-19 pandemic. The high numbers of confirmed cases and deaths have continued unabated since the first reported case, with no flattening or downward turn in the curve. In this context, healthcare workers have been exposed uninterruptedly to stress factors throughout a year of the pandemic. The study´s aim was to identify and analyze healthcare workers´ perceptions of their feelings and concerns that have surfaced in responding to the pandemic. Method: This was a cross-sectional online qualitative survey study of 554 healthcare personnel working in the state of Rio de Janeiro during the COVID-19 pandemic. Recruitment occurred from July 20 to September 30, 2020, using an online survey, preceded byfree informed consent term. Data were analyzed with the Iramuteq software. Results: Through a dendrogram, the words with the highest chi-square were highlighted and grouped into four classes: healthcare workers´ fear of falling ill to COVID-19 and infecting their family members; work/labor issues; feelings of powerlessness and need for public policies for government action; and fatigue and burnout in the pandemic. Each word class was also illustrated by a similarity tree. Conclusion: The study revealed healthcare workers´ exacerbated fear of infection and transmission of COVID-19 to their family members, besides financial losses and feelings of powerlessness and abandonment.

## Introduction

The COVID-19 pandemic has produced a persistently number of cases with moderate and severe clinical conditions that often require admission to hospital wards and/or the Intensive Care Unit (ICU) [1]. The impact for administrators and healthcare workers has raised huge challenges for the population´s healthcare. The World Health Organization (WHO) has declared that healthcare workers are exposed to greater risk of mental and psychiatric disorders. The healthcare workers are more exposed to biological risk, even if they can access adequate training and equipment for providing [2].

At the beginning of the pandemic, the intense exposure of health professionals to biological risks due to lack of material and human resources [3, 4]. Knowledge of the disease was limited and specialized equipment insufficient, which contributed to the severity of this first phase [5]. Everything needed to be “reinvented”. The fear of getting sick, of developing severe forms of the disease and taking the virus to the family was a concern, but fear still persists among health professionals, and has profoundly changed the social and work environments, being a major generator of anxiety and other symptoms related to mental health [4].

Like other countries, Brazil has suffered the impacts of COVID-19. The first reported case was on February 26, 2020, and since then the incidence and mortality have continued to grow and fluctuate [6], making a second wave identification difficult once there was no record of a significant drop before. On September 2, 2020, the World Health Organization registered 570 thousand professionals infected with COVID-19 and 2.5 thousand died in the Americas [7] corresponding to the largest number of health professionals infected in the world. Until February 22, 2021, 125,046 cases of Respiratory Syndrome suspected of COVID-19 had been reported in health professionals in the “e-SUS Notifica” [8]. Of these, 33,453 (26.7%) were confirmed for COVID-19. In Brazil, in this same period, the professional categories with the highest number of confirmed cases were technicians / nursing assistants (9,969; 29.8%), registered nurses (5,785; 17.3%), physicians (4,035; 12.1%) and dentists (1,498; 4.5%), the national data on the involvement of health professionals are frightening [8]. The pandemic persists with alarming cases, overloading the health system and its professionals [8]. In view of the health crisis triggered by SARS-CoV-2, and the persistence of the pandemic for just over a year, in June 2021, high rates of cases (17,210,969 confirmed cases) and deaths were still observed in Brazil (482,019 accumulated deaths) [8].

Measures to contain and prevent infection such as washing hands, mask-wearing, and social distancing, among others, have not been completely adopted by the entire population or enforced unequivocally by government authorities. The increase in the demand for healthcare services, alongside a heavy overload on the health system [1, 9] and the loss of control over events are thus potential factors generating feelings of vulnerability and fear among healthcare workers, with the potential for serious psychological and cognitive consequences [2].

The identification and assessment of stress-related factors in healthcare workers can help meet the needs of these workers and allow the implementation of measures for improvement, not only in their quality of life [10, 11], but also in their work performance. Emotional exhaustion can lead to serious errors in patient care and even jeopardize patient safety [9]. The current study aimed to identify and analyze healthcare workers´ perceptions of the feelings and concerns that have surfaced in the response to the COVID-19 pandemic.

## Method

### Study design, participants and study setting

This was a cross-sectional and qualitative study with data collection by online survey, of data from the research project clinical-epidemiological and psychosocial profile of healthcare workers in the COVID-19 pandemic in the state of Rio de Janeiro (COVIDPRO), which collected data on healthcare workers (social workers, biologists/biotechnicians, registered nurses, pharmacists, physical therapists, speech therapists, physicians, nutritionists, psychologists, nurse technicians or assistants, radiology technicians, occupational therapists, laboratory technicians, dentists) in the state of Rio de Janeiro on demographic and work characteristics as well as the workers´ subjective manifestations in the COVID-19 pandemic.

The COVIDPRO research project was conducted at the initiative of a group of researchers from the Fernandes Figueira Institute/FIOCRUZ (IFF/FIOCRUZ), a technical unit of FIOCRUZ dedicated to research, teaching, and patient care for children, adolescents, and women. The group realized the need for a better understanding of the reality experienced by these healthcare workers in Rio de Janeiro, especially those working on the frontline of care in the COVID-19 pandemic.

### Data collection procedure

Data were collected from July 20 to September 30, 2020, using an online form developed in Google Forms, consisting of 30 closed questions and one only open question. The analyzes performed by the iramuteq software included only the data from the open question: “We would like to leave this space open for you to write how you feel at this moment in the pandemic or make suggestions on questions that were not covered in this study”. The closed questions were related to sociodemographic, professional and performance data in the COVID-19 pandemic of health professionals, but for a qualitative analysis of the data that generated this article, it only uses the open question of the questionnaire. Participants were only allowed to complete the form after reading and confirming the online informed voluntary consent form.

### Inclusion and exclusion criteria and sample size

**Fig 1:**
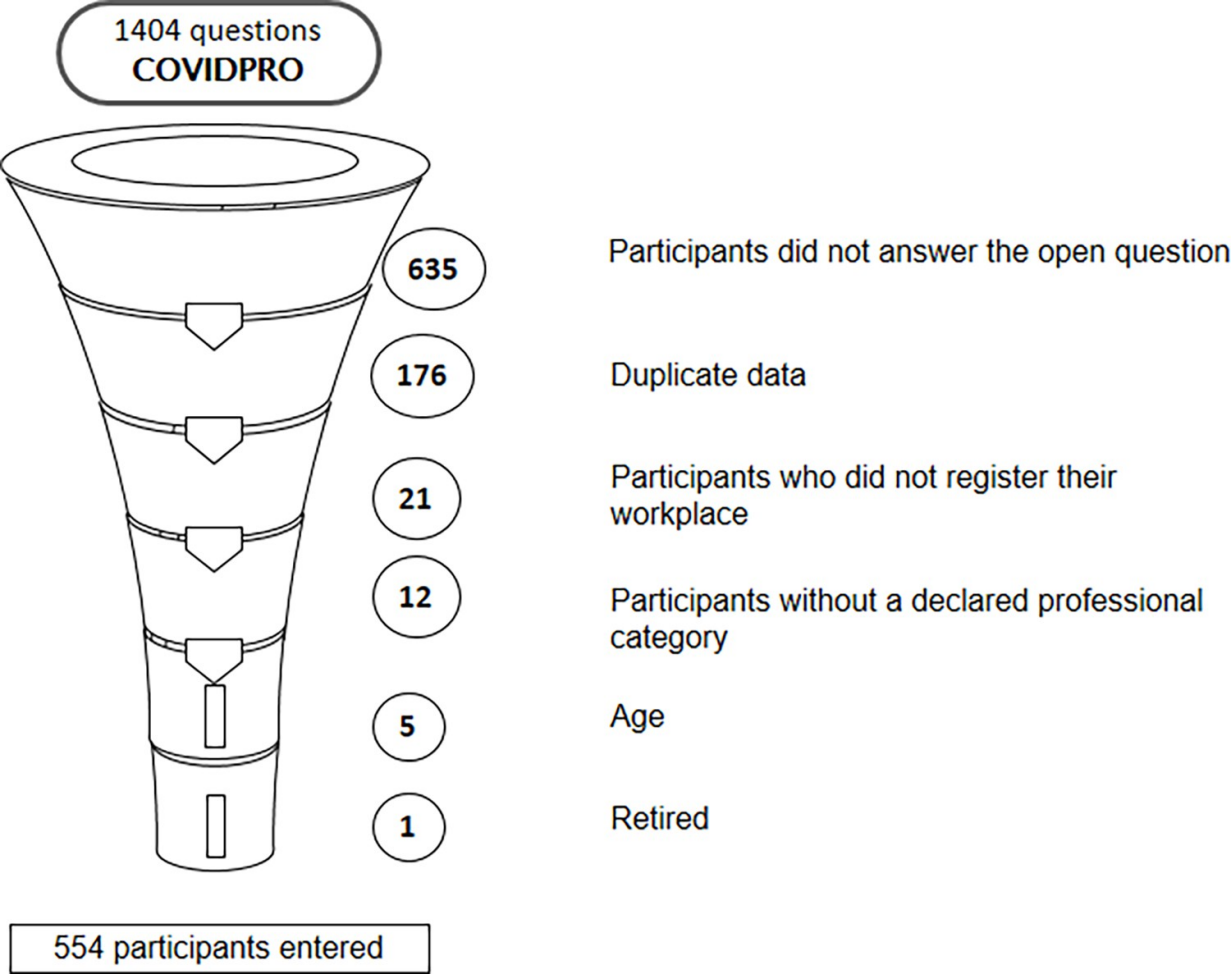
Participant recruitment diagram. Source: COVIDPRO Project (2020). Rio de Janeiro–Brazil, 2020. (N = 554).

Data were collected using convenience sampling methods. The study sample consisted of all the records of responses by healthcare workers who completed the open item of the online questionnaire. Inclusion criteria: being a health professional, working during the COVID-19 pandemic, in the state of Rio de Janeiro, over 18 years old and responding to the open field of the data collection instrument. Exclusion criteria: retired healthcare workers or those with incomplete data on age, sex, and professional category, since these variables were used in composing the line of command in the Iramuteq data analysis software. The study had 1404 participants, but 850 were excluded for the reasons presented in Fig 1, with this, 554 participants were included.

### Variables

The following variables were analyzed: professional category, age (stratified18 to 39 years, 40 to 59, and 60 or older), sex (male and female), and the excerpts of text recorded in the open field.

### Development

The segments of text were analyzed with the software Iramuteq (*Interface de R Pour Les Analyses Multidimnsionnelles de Textes et Questionnaires*), which allows executing various analyses of textual data, such as calculation of frequency of words or multivariate analyses. The tool is also capable of organizing the words´ distribution so as to facilitate understanding and viewing the data through analysis of correspondences, analysis of similarity, and word cloud [12, 13]. The analyses performed by this software allow organizing the data obtained in a dendrogram that illustrates the relations between classes and describes the principal results. The dendrogram displays the partitions or interactions that were performed in the classification of the text segments (TS) in the corpus [12].

The following steps were elaborated in this study for the data analysis in Iramuteq: *stage 1– composition of the corpus; stage 2 –elaboration of the lines of command; stage 3 –correction and revision of the corpus; stage 4 –lexical analysis; stage 5 –textual analysis according to the Reinert method; and stage 6 –analysis of similarity*.

#### Stage 1—Composition of the corpus

This stage consists of the composition of the corpus, which is the set of texts from the various participants, formed by the TS that comprised the answer to the open question. It is necessary to transport all the participants´ texts (answers to the single open question) to a single file in UTF-8 format, leaving the first line blank.

#### Stage 2—Elaboration of the lines of command

This stage consists of defining the lines of command, which allow separating the textual material submitted by each of the participants. In this study the lines of command were defined by the variables: professional category, sex, and age. In the text file in UTF-8 format, we inserted at the beginning of the text the participant´s identification and characteristics in the following sequence: **** *id_participant *professional_category *sex *age, where id_participant is a numerical sequence starting with 001.

#### Stage 3—Correction and revision of the corpus

This is the most labor-intensive and painstaking stage. The first step of this stage is to perform the text´s revision and correction (correcting keying-in, spelling, and grammatical errors, besides excluding any special symbols or characters, such as “%”, “"”, “*”, “&”, “$”, “-” etc.). The second step is to standardize the numbers, terms, and abbreviations. Finally, in the third step it is necessary to define for Iramuteq which sets of words form a compound term (for example: Ministry of Health, which should be referenced in Iramuteq as Ministry_of_Health).

#### Stage 4—Lexical analysis

This stage consists of importing the data from the corpus of the UTF-8 file to Iramuteq. This requires selecting the text file and selecting the language that will be evaluated. Iramuteq version 0.7.alpha.2 works with five languages (English, French, Italian, Portuguese, and Spanish) and allows defining the standard dictionary to be used to identify synonyms in the selected language. For the current study, the corpus was analyzed with Portuguese as the principal language.

#### Stage 5—Analysis of the text according to the Reinert method

The text was analyzed with the Reinert method, which suggests a downward hierarchical classification (DHC) that aims to obtain TS that present similar vocabulary to each other, and different vocabulary from the TS of the other classes, as occurs with hierarchical grouping analysis [12]. The TS are classified as a function of their respective vocabularies, and the whole set is partitioned as a function of the presence or absence of the reduced forms [14]. Based on the chosen classes, the classification algorithm calculates and furnishes the most characteristic TS in each class. This analysis is performed with the chi-square (degree of freedom) [12, 15]. DHC-type analysis requires minimal retention of 75% of the TS to be considered useful. The DHC algorithm allows organizing the data in a dendrogram that illustrates the relations between classes and describes the main results. The dendrogram displays the partitions or interactions performed in the classification of the TS in the corpus [12].

In DHC, Iramuteq processes the text in such a way as to allow identifying the word classes and thus to allow inferring which idea the textual corpus intends to transmit. Each class consists of a group of words with distinct frequencies, but with the highest x^2^, meaning stronger association between these words per class. By reading the TS corresponding to these words, it is possible to identify common elements within each class and thereby define a nomenclature that symbolizes each class. The classes received headings according to the mean of their TS and will be presented according to their division in the dendrogram.

#### Stage 6—Analysis of similarity

Analysis of similarity is based on graph theory and allows identifying co-occurrences between the words, with the result providing indications of connexity between words, assisting the identification of the structure in a textual corpus, also distinguishing the common parts and specificities as a function of the illustrative (descriptive) variables identified in the analysis [16].

### Ethical approval and consent to participate

The research project is registered in Platform Brazil under CAAE: 31997320.5.0000.5269 and approved by the Institutional Review Board of IFF/FIOCRUZ under case review 4.102.925. All the participants electronically signed an online consent form.

## Results

A total of 1,190 replies to the online survey were obtained. Of these, 554 were selected, having completed the open-ended item and met all the study´s eligibility criteria. The characteristics of the participants included in the study were displayed in Table 1.

**Table 1:** The characteristics of the participants included in the study.

Characteristic of Professions	(n = 554) Number (%)
**Sex**	
Female	473 (85,38%)
Male	81 (14,62%)
**Age bracket (years)**	
18–39	151 (27,26%)
40–59	305 (55,05%)
≥ 60	98 (17,69%)
**Professions**	
Physicians	219 (39,53%)
Nurses	115 (20,76%)
Nurse technicians or assistants	67 (12,09%)
Physical therapists	43 (7,76%)
Psychologists	36 (6,50%)
Dentists	12 (2,17%)
Other professional categories	62 (11,19%)

### Downward hierarchical classification

**Fig 2:**
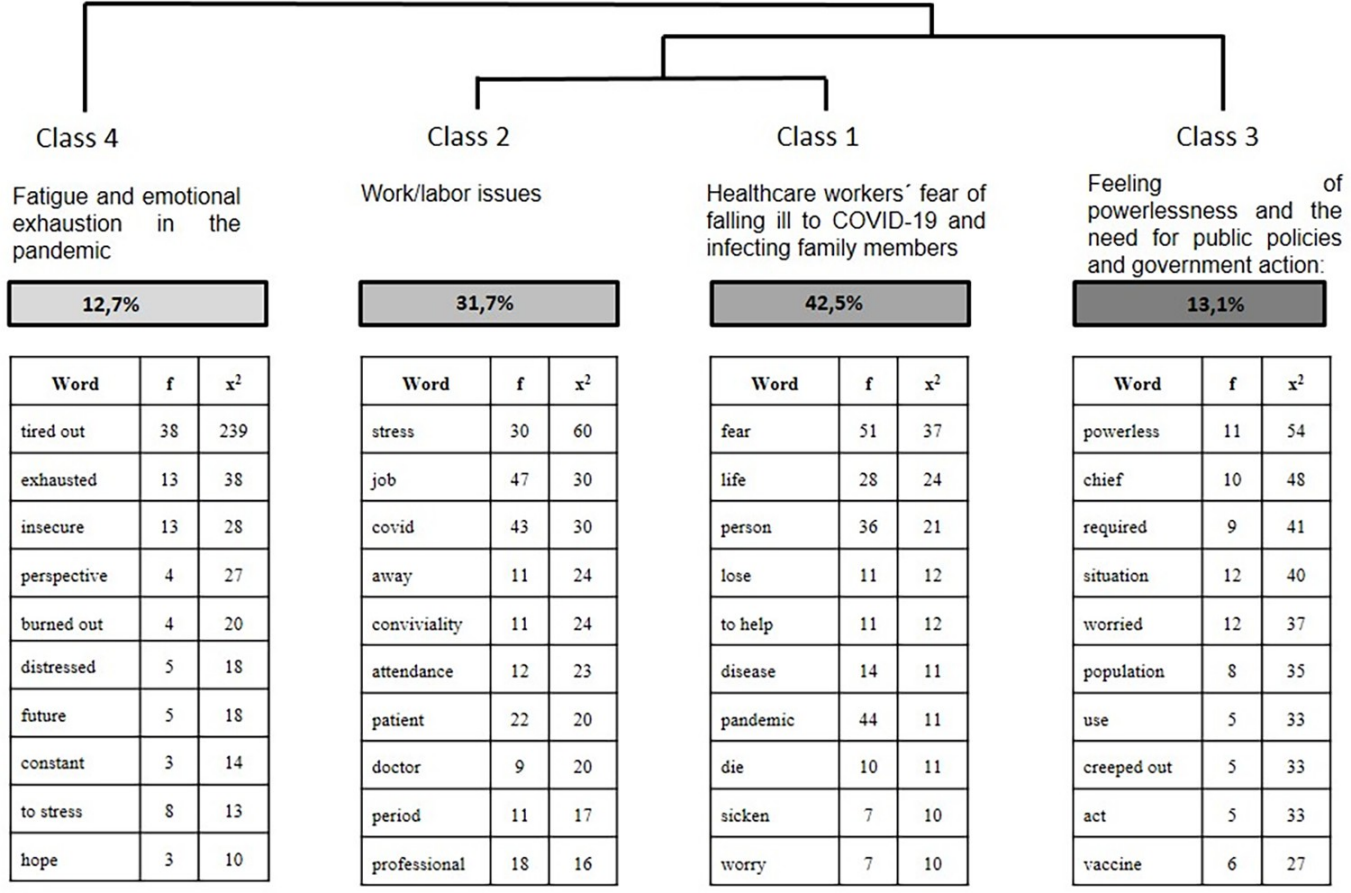
Dendrogram of downward hierarchical classification of classes. Source: COVIDPRO Project (2020). Rio de Janeiro–Brazil, 2020. (N = 554).

The classes received headings according to the meaning of their TS presented according to the dendrogram´s division Fig 2. The dendrogram thus obtained: **Class 1:** Healthcare workers´ fear of falling ill to COVID-19 and infecting family members, corresponding to 42.48% of the TS and 240 words; **Class 2:** Work/labor issues: totaled 31.68% of the TS, with 179 of the analyzable words; **Class 3:**Feeling of powerlessness and the need for public policies and government action: totaled 13.1% of the TS, with 74 words; and **Class 4:** Fatigue and emotional exhaustion in the pandemic: classified 72 words in class 4, with 12.7% of the TS.

### Class 1: Healthcare workers´ fear of falling ill to COVID-19 and infecting family members

Class 1 accounted for 42.5% of the TS, with the following main elements: fear (x^2^ = 37.0; p<0,0001), life (x^2^ = 21.5; p<0,0001), person (x^2^ = 21.0; p<0,0001), loss (x^2^ = 12.1; p = 0,000049), help (x^2^ = 12.1; p = 0,00073). The healthcare workers mainly expressed fear associated with the risk of being infected with the virus and developing a severe case of the disease, as well as exacerbated fear of transmitting the disease to their family members, as shown in the following excerpts:

*“afraid of contracting [the virus] and developing a more serious form and dying*, *or even worse*, *transmitting it to my children*, *mother*, *or husband*, *or even friends*, *with them developing serious cases and dying” (Nurse*, *female*, *45 years*).

“*I´m afraid of infecting my mother and of her developing a serious case*. *How could I save so many lives from COVID and lose my 52-year-old father*, *one of the most important lives*, *always by my side*? *I gave him the right treatment*, *but it was not enough*. *I don´t feel bad*, *thinking I could have given him more medication*, *but I feel bad for him*, *having died when I saved so many others at greater risk than him” (Physician*, *male*, *23 years)*.

In this context of the pandemic, many healthcare workers voiced thoughts on the impact on life, as expressed in the following excerpt.

“*I´ve been in the health field for nearly 20 years*, *and I´d never felt so abandoned*, *with no information*, *and so vulnerable*. *Never needed antidepressants*. *My professional life had never affected my personal life” (Nurse technician*, *female*, *41 years)*.

**Fig 3:**
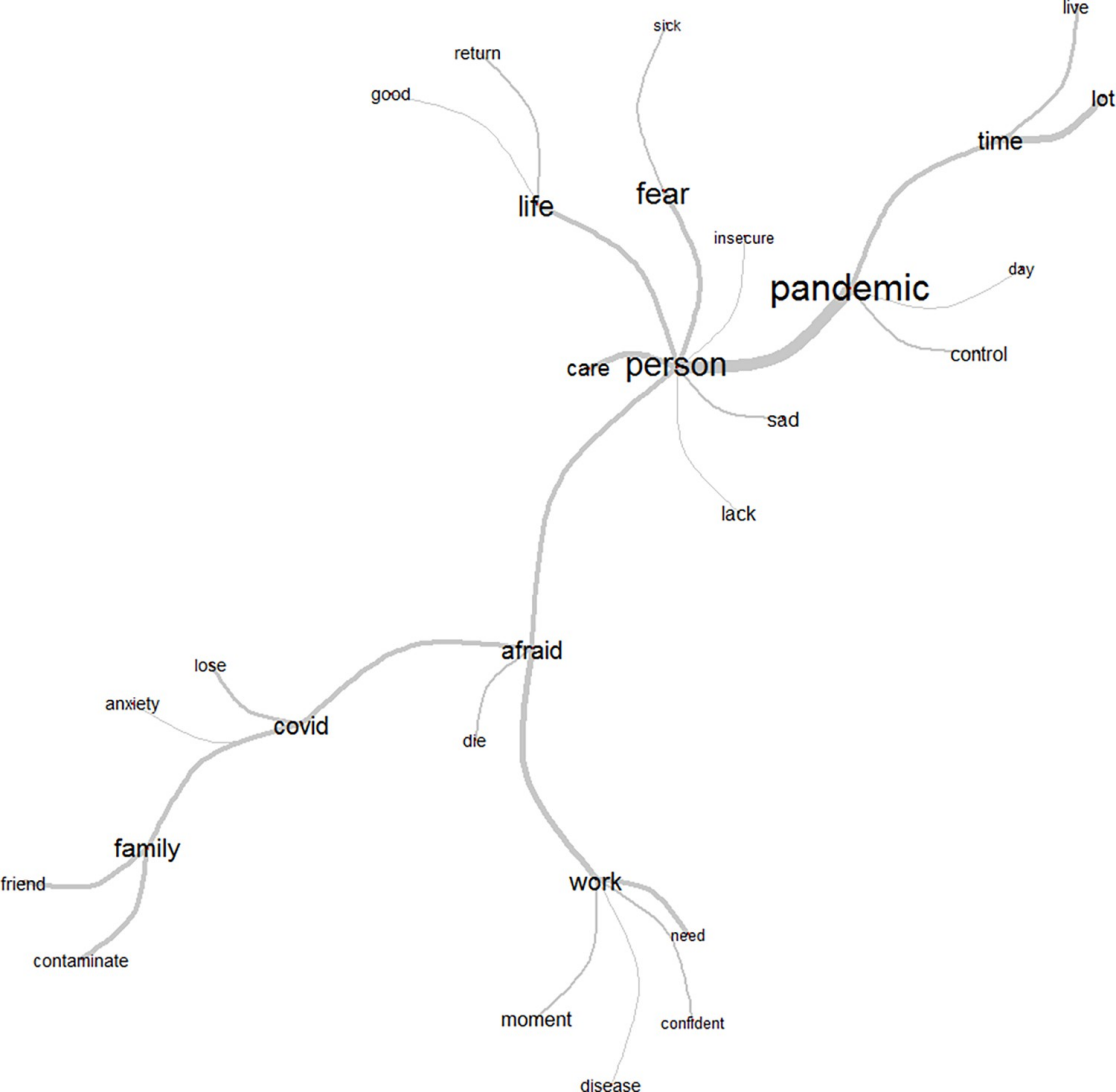
Similarity tree of Class 1: Healthcare workers´ fear of falling ill and infecting family members and friends. Source: COVIDPRO Research Project (2020). Rio de Janeiro–Brazil, 2020. (N = 554).

Fear, pandemic, and person were the main words representing class 1, observed in the similarity tree Fig 3. There was a strong connection between the word fear and family, children, friends, transmit, COVID, and die, reflecting the fear of infection and transmission. The other extremity in this branch centers on the word pandemic, which relates to the feeling of sadness, anxiety, help, and control, connoting the relations established during spread of the virus. The connection between the extremities of this tree consists of the word person, referring to the healthcare worker´s own life, establishing a connection between fear and the pandemic.

### Class 2: Work/labor issues

Class 2 presented 31.68% of the TS, with the following principal elements: stress (x^2^ = 60.3; p<0,0001), work (x^2^ = 30.3; p<0,0001), COVID (x^2^ = 29.9; p<0,0001), removal (x^2^ = 24.1; p<0,0001), and contact (x^2^ = 24.1; p<0,0001).The most emphatic feeling in this class was work-related stress:

“*I feel overburdened*, *discriminated*, *and blocked from my coworkers due to my profession” (Nurse*, *female*, *35 years)*.

“*Even with all the care that the COVID situation requires with myself*, *my family*, *and patients*, *the Ministry of Health fired me and my coworkers*. *I had this job*, *where the contract did not mention dismissal*. *The nervousness from unemployment contributed even more to the stress” (Nurse technician*, *female*, *49 years)*.

“*Vacations and holidays were suspended at this difficult moment of stress and loss of coworkers*. *Labor rights were suspended*, *like time-on-the-job bonus pay*, *bonus leave*, *no time-and-a-half pay for night shifts*, *for coworkers on leave due to preexisting conditions or COVID” (Nurse*, *female*, *51 years)*.

“*I often feel depressed because I´m on leave from my workplace at the Institution*, *since I´m part of the risk group” (Physician*, *male*, *76 years)*.

“*My son is four years old*, *and life with him is really hard now*, *with school closed*, *and he gets upset and agitated because I´m gone so long*, *and when I come home*, *he lets out all the resentment he feels over my absence” (Physician*, *female*, *40 years)*.

**Fig 4:**
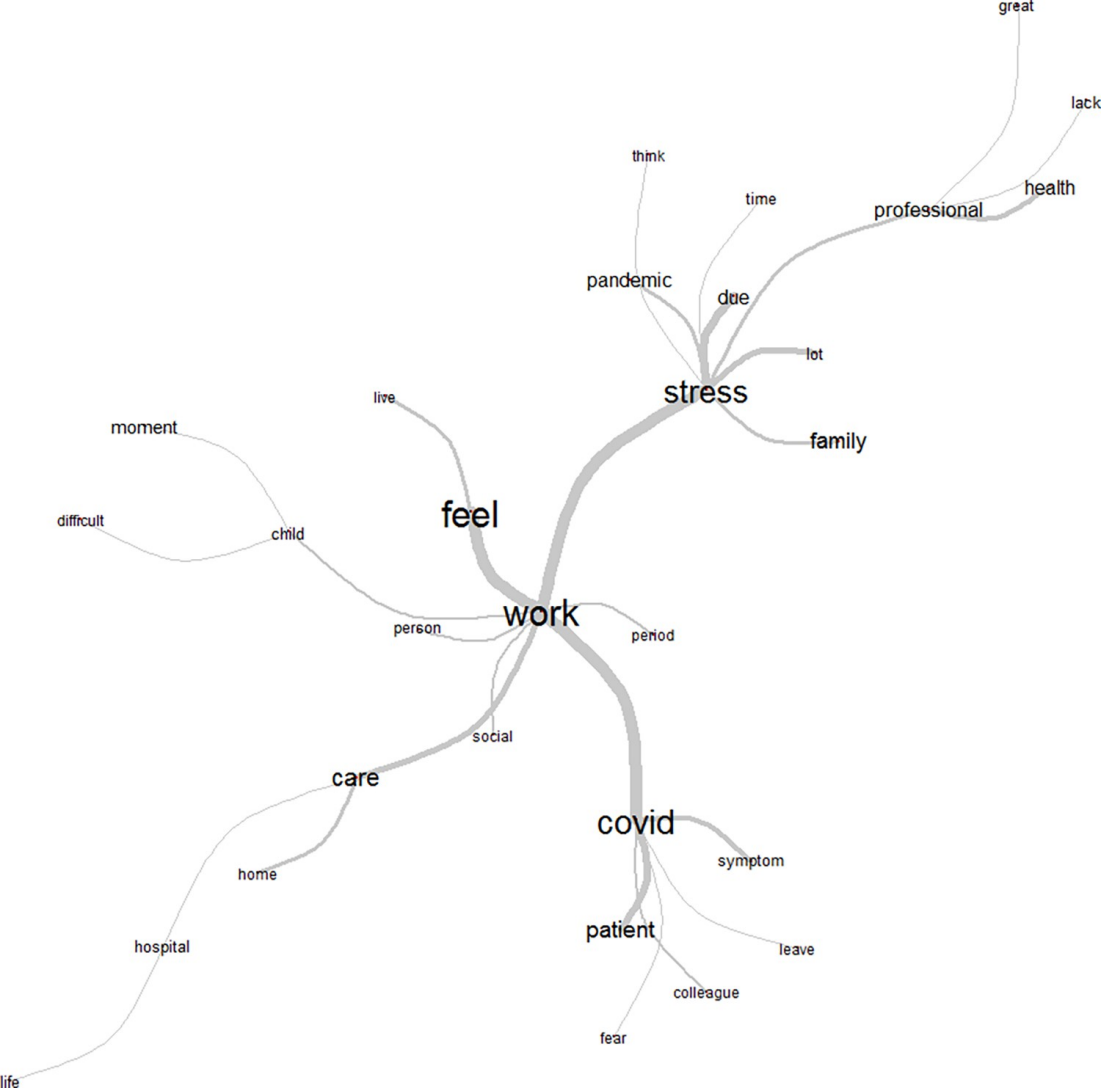
Similarity tree for Class 2: work/labor issues. Source: COVIDPRO Project (2020). Rio de Janeiro–Brazil, 2020. (N = 554).

Analysis of similarity in class 2, represented in Fig 4, shows a strong connection with the word work, with four peripheral axes. Thus, work is related to feeling a lack of contact, and of being removed. There was also a relationship between the word work and stress, reflecting the moment and the healthcare worker´s activity. There was also a connection between work and children, connoting the idea of difficulties faced in this area, mainly due to the need to be away from home. Finally, it reflects the relationship to the word COVID, indicating a link to the patient, symptoms, hospital environment, coworkers, and fear.

### Class 3: Feeling of powerlessness and need for public policies and government action

This class accounted for 13.1% of the TS, with the following main elements: powerless (x^2^ = 54.0; p<0,0001), authorities (x^2^ = 47.6; p<0,0001), necessary (x^2^ = 41.2; p<0,0001), situation (x^2^ = 40.0; p<0,0001), worried (x^2^ = 37.1; p<0,0001). The healthcare workers´ quotes point to concern and dissatisfaction over the lack of support from the institution´s administrators and the municipal, state, and federal authorities:

“*powerless and sad because of ineffective and corrupt health policy at the federal*, *state*, *and municipal levels*, *denialism*, *negligence towards the population” (Physician*, *female*, *60 years)*.

“*I feel that the situation with the pandemic in the country has been out of control for many months*, *and that the population has not received sufficient clarification from the three spheres of government*, *while there´s a lot of misinformation” (Physician*, *female*, *64 years)*.

“*worried about the governmental chaos the country is experiencing and the countless deaths that could have been avoided” (Psychologist*, *female*, *71 years)*.

“*at this moment when the number of cases has decreased considerably*, *I feel more at ease*, *confident that the situation is under control*, *little by little” (`Physician*. *female*, *29 years)*.

“*I´m sad to see the people disrespecting* [social distancing] *or failing to take the pandemic seriously*. *My own father is part of the statistics*, *so I know from personal experience what COVID can do” (Physical therapist*, *female*, *46 years)*.

**Fig 5:**
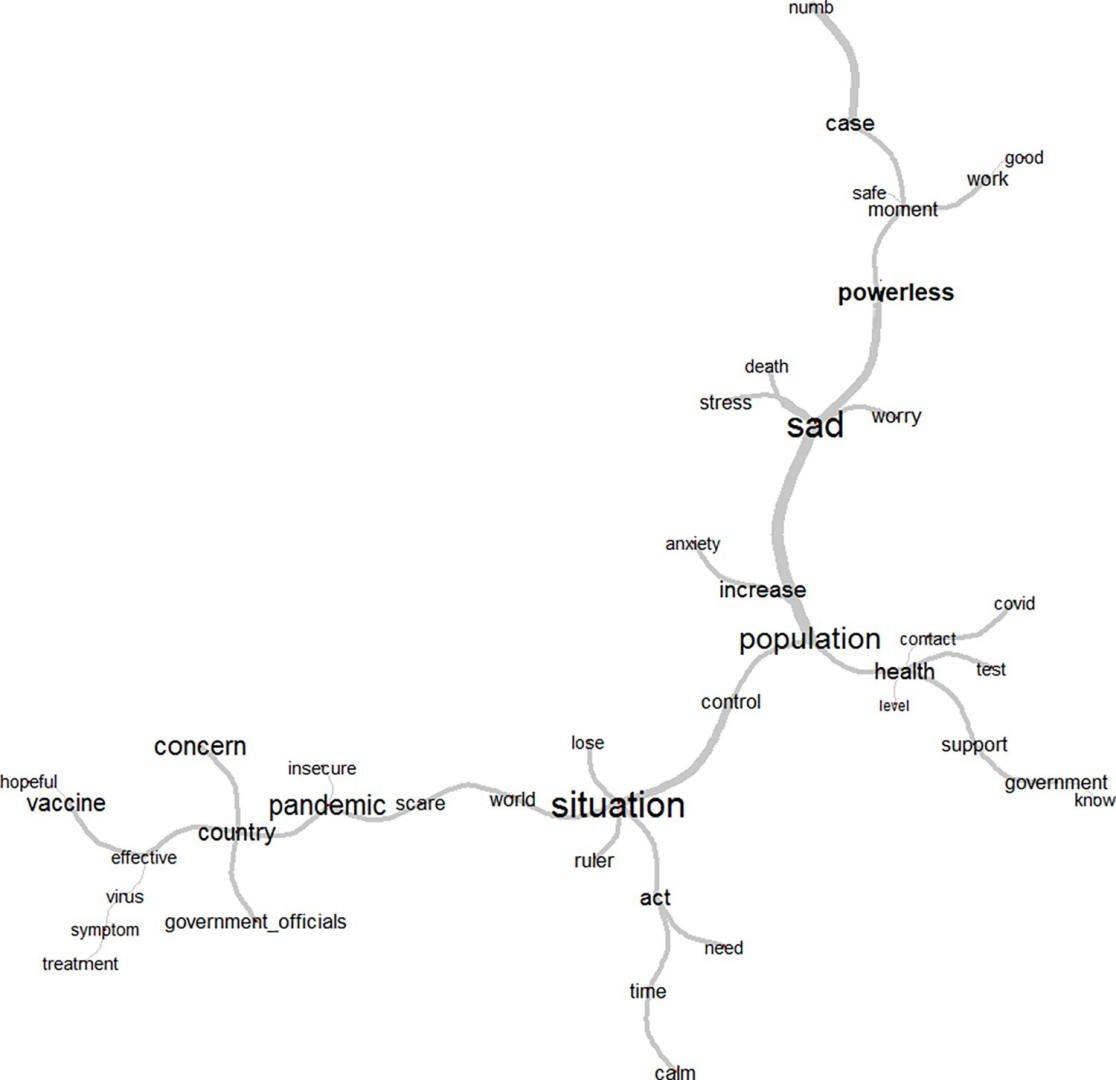
Similarity tree for Class 3: feeling of powerlessness and need for government policies and action. Source: COVIDPRO Project (2020). Rio de Janeiro–Brazil, 2020. (N = 554).

The analysis of data similarity in class 3 generated a tree that expresses intense connection between the word powerless and the feeling of sadness Fig 5. There is a connection from the central axis to the peripheral branch to the word necessary, traversing the words vaccine and hope. Another important branch reproduces sadness with the situation of worrying, mentioning the population´s health and actions by the country and the authorities. There is another peripheral branch of persons referring to feeling more at ease, with fewer cases of COVID-19. This tree refers to the feeling of powerlessness in the face of government acts and generating feelings of concern and at the same time a search for hope, glimpsed in the vaccine.

### Class 4: Fatigue and emotional exhaustion during the pandemic

This class accounts for 12.74% of TS, with the following principal lexical structures: tired (x^2^ = 239.4; p<0,0001), exhausted (x^2^ = 38.1; p<0,0001), insecure (x^2^ = 28.3; p<0,0001), perspective (x^2^ = 27.5; p<0,0001), and burned-out (x^2^ = 20.5; p<0,0001).

The participants´ quotes revealed negative elements, both physical and emotional strain, related to professional practice. Frontline healthcare workers highlighted fatigue, burnout, and physical exhaustion from the pandemic, expressed in the following excerpt:

“*I feel tired and stressed because I work nonstop and I can´t see the end of the pandemic and a rebeginning with a new normal” (Physician*, *female*, *49 years)*.

“*Abandoned*, *helpless*, *disrespected*, *exhausted*, *depressed*, *hopeless*, *discouraged*, *indebted*, *I went two months without receiving my main source of income*, *humiliated” (Physician*, *female*, *49 years)*.

“*I´ve felt terrible fatigue*, *anxiety*, *and concern for the future*, *because I´ve watched out for myself and for others*, *but people have been doing exactly the opposite” (Social worker*, *male*, *31 years)*.

**Fig 6:**
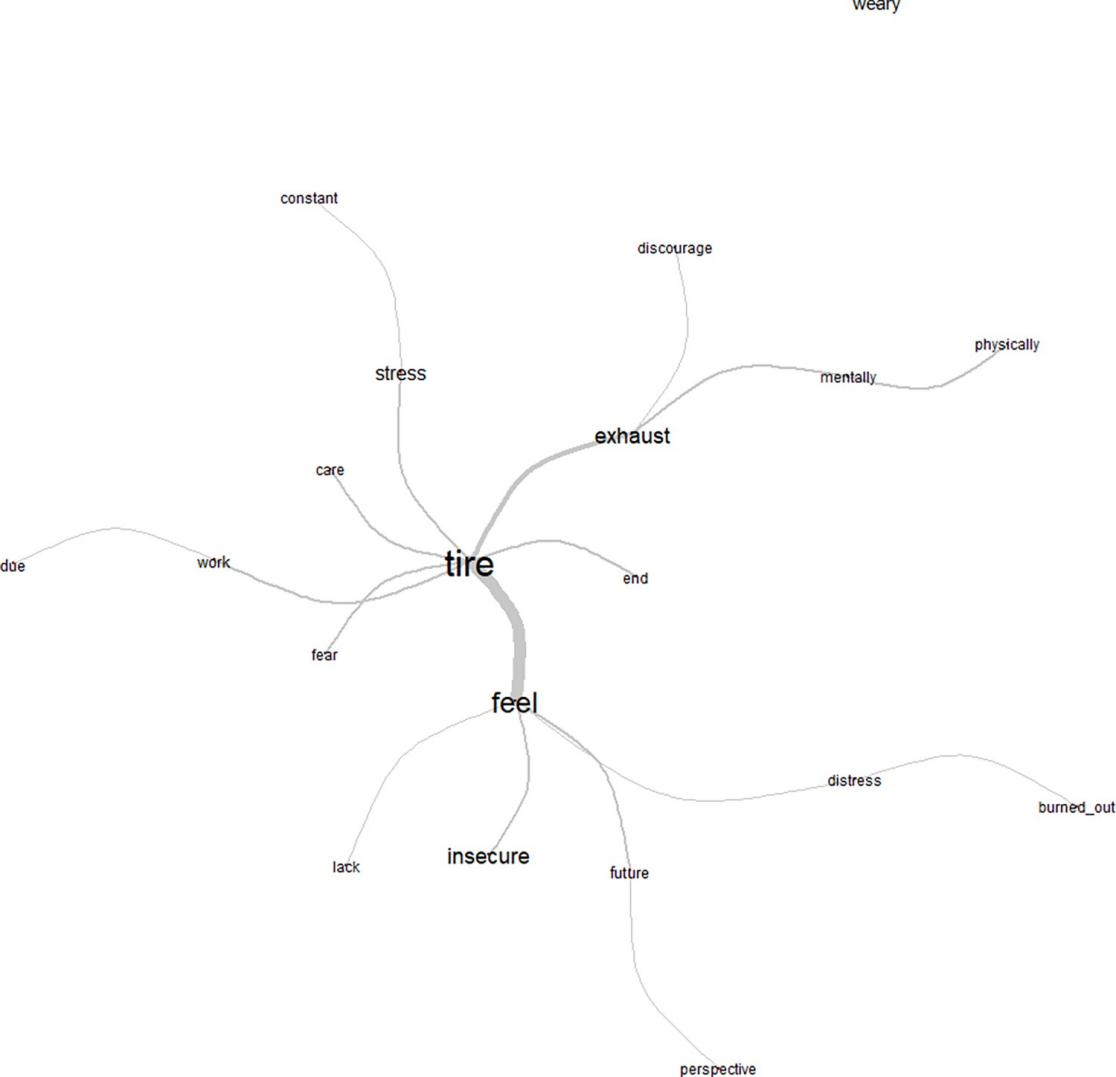
Similarity tree for Class 4: Fatigue and burnout in the pandemic. Source: COVIDPRO Project (2020). Rio de Janeiro–Brazil, 2020. (N = 554).

The similarity tree generated for class 4 showed the central word tired, which obtained chi-square (degree of freedom): 239.4, the highest chi-square evoked in the open responses Fig 6. The word tired relates to fear, stress, overload, hope, exhausted, burnout, and a feeling of insecurity about the future, a need for prospects for the future.

## Discussion

This study showed that, facing the pandemic generates in health professionals feelings of insecurity, fear, worry, professional devaluation, lack of motivation, stress, impotence, exhaustion.

It is essential for healthcare workers to feel that their families are safe, and that society values their work by adopting precautionary measures against COVID-19, for these workers to be able to embrace their difficult task with courage and hope [17]. In addition to fear of infection, the study revealed a pervasive feeling of professional devaluation.

The results highlighted fear of being infected with SARS-CoV-2 and transmitting the virus to the family as the predominant feelings in class 1, with the highest chi-square. This concern over falling ill and infecting one´s family was observed in previous epidemics, including H1N1 influenza A (2009) and SARS-CoV-1 (2003) [4]. This concern has a basis in fact, since in September 2020, the Pan-American Health Organization reported that there had been more than 570 thousand COVID-19 cases and 2,500 deaths among healthcare workers in the Americas [6]. Lack of personal protective equipment (PPE) and adequate training, mainly at the beginning of the pandemic, may have contributed to this tragic outcome [7]. According to an online survey of Brazilian healthcare workers in the first semester of 2020, the majority had not received appropriate training for treating COVID-19 patients [18]. During the initial months of the pandemic, overburdened healthcare services forced healthcare workers to deal with prolonged shifts, fatigue, burnout, and lack of PPE or of adequate training. In this context, preventive measures and supervision of practices are important for protecting healthcare workers and their families [19].

The work/labor issues that appeared in class 2 highlighted different experiences depending on the health professional’s situation. Among the health care workers on the frontline, issues such as being away from social life appear as an act of protection of family and friends but also due to discrimination for being health professionals. Among those on work leave because they belonged to the risk group, there were reports of discomfort and dissatisfaction. This was also seen in a study showing that employees under quarantine experience a conflict with their roles as healthcare workers and parents/family caregivers, feeling guilty for having left the frontline at a time of workforce shortage and at the same time afraid of being infected or infecting their families [20]. Financial instability, wage cuts, worrisome levels of job insecurity, devaluation, and loss of labor rights at the height of the epidemic were also recorded as stress factors mentioned in other studies [21, 22]. This instability and financial losses were observed among those on work leave due to the loss of bonuses, but also among frontline health care workers that have been harmed by temporary employment contracts suffering from late payments and sudden dismissals. The suspension of vacations was another factor that triggered stress among healthcare workers. Another challenge that healthcare workers have faced is the issue of childcare, especially because schools have been closed for a long time in Brazil, and many elderly persons are part of the support network that healthcare workers need to avoid contact with, making care for their children more difficult and complicated [23].

Work-related stress in the pandemic was a major factor in class 2. This feeling was interwoven with others that impacted emotional life and interfered directly in the quality of life of these workers. In Brazil, stress has been continuous and uninterrupted in a public health context that was already unsatisfactory for healthcare personnel before the pandemic. The global health emergency revealed the cycle of reproduction of poverty and social iniquities laying bare, in Brazil, the historical neglect towards public policies, including workers´ devaluation [17, 24]. The pandemic has displayed healthcare workers´ serious vulnerability such as exposure to external factors like biological, economic, and structural risks, in addition to individual factors [17].

Healthcare workers´ feeling of powerlessness and anxiety, and of the need for public policies and government action, were shown in class 3. Major concerns include the lack of institutional support and the stance by certain government authorities and state officials who have underestimated the pandemic´s severity, contributing to the spread of COVID-19 and to a feeling insecurity, anxiety, and lack of prospects for a brighter future [21, 25]. In the midst of denialist speeches by government authorities, we found people who already had a feeling of recrudescence of the pandemic, although the number of cases was still quite high [26]. The similarity tree for this class showed a connection between the feeling of powerlessness and the word vaccine, appearing as an essential measure for immediate intervention [27].

References to unappreciation and lack of prospects for the future were common in the answers. Importantly, the study was conducted in the state of Rio de Janeiro, where a series of scandals rocked both the state and the city during the pandemic: corruption and changes in governors and state health secretaries [28]. Thus, when frontline healthcare workers most needed support and strengthening of government agencies, they felt the most unprotected and insecure, aggravated by payroll delays and layoffs [29]. The governments´ difficulty in organizing public policies to prevent crises or mitigate their consequences can be understood from an economic perspective, among other factors: disparities between economic interest groups and the general public [30]. The lack of a solid, organized policy to deal with the crisis merely aggravated the chronic abandonment, since healthcare workers felt no sympathy or support from the government, reinforcing the perception that the epidemic was out of control [31].

Class 4 emphasized fatigue and emotional exhaustion during the pandemic. The word tired was related to fear, stress, overload, hope, exhaustion, and burnout. The preexisting overload on the public health system was drastically exacerbated by the COVID-19 pandemic, intensifying the crisis situation already assailing the world population [32], generating a cycle of discontent and burnout in healthcare workers. This problem may be related to the growing number of SARS-CoV-2 cases in Brazil, leading to greater need for patients´ hospitalization and thus a heavy work demand on healthcare workers, resulting in difficulties with human resources, profound changes in the workday, and an increase in the pace and performance of overtime work [2].

The main limitations to this study were the predominance of physicians and nurses among the health professions, preventing stratification by professional category, which would have allowed more in-depth knowledge of the pandemic´s impact according to job activity. Another limitation to be considered is that, as this is a qualitative and online survey, the researchers are unable to clarify the participants’ responses in detail. The proportionally higher response by these two professions may be explained by selection bias, since the questionnaires were circulated via the social networks of the researchers, who are predominantly physicians and nurses. Another limitation was the predominance of responses by women, although this might be expected since some 70% of healthcare and social services teams in Brazil currently consist of women [33, 34]. Another limitation was the process of composing the corpus, since this stage is performed manually and is subject to errors that can interfere directly in the result obtained with Iramuteq. To minimize the occurrence of these errors, this stage was performed with double-checking by two researchers, and divergences were discussed and resolved in a consensus group. Even with these limitations, one of the key strengths of the study is that the Iramuteq software expands the textual analysis to a large dataset beyond simple frequencies.

## Conclusion

The study revealed healthcare workers´ exacerbated fear of being infected with SARS-CoV-2 and transmitting the infection to their families, besides financial losses and feelings of powerlessness and abandonment. These feelings were aggravated by lack of political direction or of implementation of effective measures to minimize and control the spread of the disease. Some of these healthcare workers manifested signs of physical and emotional illness, evidencing a second, silent pandemic of negative feelings and heavy stress.

The analysis also revealed the workers´ prolonged exposure to stressful factors such as work overload and fatigue. Healthcare workers´ precarious work conditions, which existed before the pandemic, have been exacerbated during the COVID-19 pandemic. These precarious conditions reveal barriers in the implementation of public policies to protect health workers´ own health.

For these healthcare workers to feel less abandoned and more secure, it is the responsibility of governments and administrators to furnish timely and accurate information based on scientific evidence. Valuing the workforce and implementing healthcare policies are also strategic actions to support the mental and occupational health of these workers.

Considering the persistence of the pandemic and consequently the continuous physical and mental overload of health professionals to stressful factors, it is valid to evaluate the reports of these professionals, to listen to their perceptions, detect warning signs and promote possible early intervention of clinical and especially emotional support during the pandemic, configuring a preventive health care measure for this professional class.

## Supporting information

S1 File (DOC)

S1 Data (XLS)
