# Supplementary material for: Repercussions of the COVID-19 pandemic on health professionals in the state of Rio de Janeiro / Brazil
Source: PLoS One. 2022 Jan 21;17(1):e0261814. doi: 10.1371/journal.pone.0261814 (PMC8782304; doi:10.1371/journal.pone.0261814)
Supplement: S1 File — (DOC) [file pone.0261814.s001.doc]

**** *ind_0010 *estr_2 *sex_2 *ida_2 *prof_3

Como profissional_de_saúde e tendo passado por um momento difícil de contaminação e internação em uti, me sinto abandonada e sem interesse público em relação a nossa classe_profissional.

**** *ind_0013 *estr_2 *sex_2 *ida_1 *prof_1

Cansada e angustiada.

**** *ind_0017 *estr_1 *sex_2 *ida_2 *prof_7

Me sinto preparada, mas preocupada.

**** *ind_0018 *estr_1 *sex_2 *ida_3 *prof_11

Estou calma e tranquila. Aguardando a vacina ser descoberta e disponibilizada pelo sus.

**** *ind_0019 *estr_2 *sex_2 *ida_1 *prof_7

Sobrecarga como nunca, por excesso de demandas de terceiros e adaptações diárias no processo_de_trabalho. Pacientes, equipe_de_trabalho, amigos, parentes. Todos, por falta de acesso em outros lugares, me procuraram com suas necessidades em saúde.

**** *ind_0026 *estr_1 *sex_2 *ida_2 *prof_7

Me sinto capaz de lidar com essa situação de pandemia desde que utilizando todos os recursos para se evitar a infecção por covid.

**** *ind_0027 *estr_1 *sex_2 *ida_2 *prof_7

Por vezes cansada por trabalho fora do horário convencional devido_à necessidade_do uso_da_internet, para a qual os setores_públicos não fornecem os instrumentos necessários.

**** *ind_0029 *estr_1 *sex_2 *ida_2 *prof_7

Já passei por todas as fases, medo, depressão, choro fácil, agora estou normal.

**** *ind_0030 *estr_1 *sex_2 *ida_3 *prof_7

Qual sua expectativa de morte_eminente. Quais providências tomou em caso de morte_eminente. Esteve sozinho ou participou de grupos de estudo e convivência. Acha que melhorou por dentro.

**** *ind_0032 *estr_1 *sex_1 *ida_3 *prof_7

Me sinto contribuindo para tratamento precoce das pessoas.

**** *ind_033 *estr_1 *sex_2 *ida_3 *prof_3

Me sinto bem. Parabéns. Pelo questionário

**** *ind_0035 *estr_2 *sex_2 *ida_1 *prof_3

Hoje estou mais_tranquilo, mas no início senti enorme tristeza achando que estava na fila_da_ morte, chorava diariamente, medo passar para a família.

**** *ind_0037 *estr_1 *sex_2 *ida_1 *prof_14

Insegura e vulnerável por estar_gestante.

**** *ind_0038 *estr_1 *sex_2 *ida_2 *prof_3

Ansiedade para que a vacina chegue.

**** *ind_0039 *estr_1 *sex_2 *ida_2 *prof_14

O medo é de contrair o vírus estando assintomática e transmitir ao meu pai e filho. Mas o isolamento_social não tem sido problema, pois sou uma pessoa que não gosta de aglomerações e prefiro estar em_casa mesmo. Me preocupa minhas amigas que estão realmente isolada e não estão bem psicologicamente.

**** *ind_0043 *estr_1 *sex_1 *ida_3 *prof_7

Tranquilo. Um dia depois do outro.

**** *ind_0048 *estr_1 *sex_2 *ida_2 *prof_4

Profissional_de_saúde não deveria se negar a usar_máscara.

**** *ind_0050 *estr_1 *sex_1 *ida_2 *prof_5

Que isso vai passar.

**** *ind_0052 *estr_1 *sex_2 *ida_2 *prof_7

Problemas família se intensificaram.

**** *ind_0055 *estr_2 *sex_2 *ida_2 *prof_5

Cansada.

**** *ind_0057 *estr_2 *sex_2 *ida_2 *prof_5

Sinto muita falta de contato_físico com meus amigos e família.

**** *ind_0058 *estr_1 *sex_2 *ida_3 *prof_7

Momento de reflexão e valorização_da_vida.

**** *ind_0060 *estr_1 *sex_2 *ida_2 *prof_3

Sinto que é uma oportunidade de crescimento enquanto sociedade, e que precisamos aprender com o que temos disponível. Tenho aplicado esta fórmula ao meu cotidiano e tem dado muito certo.

**** *ind_0061 *estr_2 *sex_1 *ida_2 *prof_7

Houve exagero na fase mais recente, uma vez que os casos estão absolutamente diminuído. O destensionamento tem que ser mais rápido. A epidemia serviu para muitas pessoas, principalmente servidores, se licenciarem sem necessidade, uma vez o que nem todo trabalho pode ser remoto.

**** *ind_0064 *estr_1 *sex_2 *ida_3 *prof_3

Tenho muito medo de me reinfectar. Não existe uma garantia que estou imunizada. Tenho muito medo de voltar a rotina_diária do trabalho e me contaminar no trajeto e no trabalho. Sinto tristeza em isolamento_social e com saudades de reunir com amigos e família.

**** *ind_0067 *estr_2 *sex_2 *ida_2 *prof_7

Nunca senti tanto medo, pensei em desistir da medicina o fator político me deixou triste e desmotivada.

**** *ind_0070 *estr_2 *sex_2 *ida_1 *prof_3

Me sinto exposta e ao mesmo tempo desvalorizada. Abro mão de ficar no meu lar e com minha família e amigos para poupar eles e também poupar meus pacientes e não sou nem remunerada decentemente. Frustrada, cansada e estressada parece nunca vai_passar.

**** *ind_0074 *estr_1 *sex_2 *ida_2 *prof_14

Ficar em isolamento_social é deprimente, como que ilhado, creio não estar mentalmente bem, terror de voltar a trabalho assinando como espontâneo, pavor de não conseguir cumprir obrigações_financeiras tendo salário_reduzido.

**** *ind_0079 *estr_2 *sex_2 *ida_1 *prof_3

Cansada, exausta, o acúmulo do home_office, de duas instituições de ensino_superior, muitas demandas, associada aos afazeres_do_lar e o cuidado com minha filha de 4_anos, tem sido muito_exaustivo. Não entendo como as pessoas têm a necessidade se fazer reuniões todos os dias, por horas. Além disso ter que conviver com contextos sociais e econômicos desastrosos, uma política indecente. Todas essas questões vão nos minando pouco a pouco. Vivemos em uma pandemia moral.

**** *ind_0084 *estr_1 *sex_1 *ida_3 *prof_7

Indignado pelo modo que o governo_federal enfrenta a pandemia.

**** *ind_0086 *estr_2 *sex_2 *ida_2 *prof_5

Cansada.

**** *ind_0089 *estr_1 *sex_2 *ida_2 *prof_7

Estou tranquila quanto ao andamento do processo.

**** *ind_0092 *estr_2 *sex_1 *ida_2 *prof_9

Nível de irritação e percepção variou muito nas diversas fases da pandemia.

**** *ind_0094 *estr_1 *sex_1 *ida_3 *prof_7

Preocupado porque as pessoas não seguem o isolamento_social nem os cuidados de prevenção o que me preocupa de retomar às atividades normais. Poderiam perguntar se os profissionais_de_saúde estão seguindo o isolamento_social como deve ser feito.

**** *ind_0095 *estr_2 *sex_2 *ida_1 *prof_3

Conseguindo controlar o estresse e ansiedade

**** *ind_0097 *estr_1 *sex_2 *ida_2 *prof_5

Habituada com os protocolos, porém ainda com bastante medo que pessoas da minha família e ou próximas venham a falecer por covid.

**** *ind_0100 *estr_1 *sex_2 *ida_3 *prof_7

Irritada por ver que muitos não estão se protegendo como deveriam e o quanto arriscam a minha_vida e a da minha_família. Entendo que a grande corrente de desinformação que exige, inclusive oficial e a falta de comprometimento dos gestores majoritários para com a população seja o maior responsável por isso mas fico incomodada.

**** *ind_0101 *estr_2 *sex_2 *ida_2 *prof_3

Ansiosa.

**** *ind_0105 *estr_2 *sex_2 *ida_1 *prof_3

O mais difícil para mim foi me isolar da minha filha no período em que positivo para o covid. Ouvir ela dizer que estava com medo u morrer foi uma dor insuportável. Uma outra coisa que me incomoda muito ainda hoje são amigos e família não fazerem o isolamento ou distanciamento_social e eu tomando todo o cuidado para não os contaminar.

**** *ind_0111 *estr_2 *sex_2 *ida_1 *prof_5

Questões com parceiros que não são da área_de_saúde.

**** *ind_0115 *estr_1 *sex_2 *ida_3 *prof_7

Com medo.

**** *ind_121 *estr_2 *sex_2 *ida_2 *prof_3

Tensa apreensiva e com medo.

**** *ind_0123 *estr_1 *sex_2 *ida_1 *prof_7

Excesso de protocolos que prejudicam o fluxo de pacientes e tratamento.

**** *ind_0126 *estr_2 *sex_2 *ida_2 *prof_7

Esgotado.

**** *ind_0128 *estr_1 *sex_2 *ida_1 *prof_14

Estou bem pois tenho um coração solidário. E acho que as pessoas são o que são, a pandemia não mudou ninguém. Quem é ruim continua ruim e quem é bom continua bom.

**** *ind_0129 *estr_1 *sex_2 *ida_1 *prof_11

Como um lixo_descartável após uso necessário.

**** *ind_0140 *estr_1 *sex_2 *ida_2 *prof_3

Sinto como uma oportunidade para recomeços.

**** *ind_0142 *estr_1 *sex_2 *ida_1 *prof_9

Me sinto um pouco triste, mas controlada e capaz de realizar minhas atividades_diárias de forma cuidado.

**** *ind_0146 *estr_1 *sex_2 *ida_1 *prof_10

Eu me sinto triste por ver o tanto de pessoas que não tem o menor cuidado.

**** *ind_0148 *estr_1 *sex_2 *ida_3 *prof_3

Procuro suporte_psicológico para me harmonizar

**** *ind_0156 *estr_1 *sex_2 *ida_2 *prof_3

Gostaria que fosse apresentado uma vacina eficaz o mais rápido possível e que aqueles que não estão seguindo as normas principalmente o uso_de_máscaras e isolamento_social fossem de alguma forma responsabilizados.

**** *ind_0160 *estr_2 *sex_2 *ida_2 *prof_7

Medo adoecer e ou que meus pais e irmãs fiquem doentes e qualquer um de nós venha a ser internado e até morrer.

**** *ind_0162 *estr_2 *sex_2 *ida_2 *prof_7

Irritada, esgotada, angustiada, sem vislumbrar melhoras rápidas.

**** *ind_0165 *estr_1 *sex_2 *ida_2 *prof_7

Me sinto já radicada de tudo isso.

**** *ind_0170 *estr_2 *sex_2 *ida_1 *prof_8

Durante a pandemia, meados de abril descobri que estava grávida. Foi um turbilhão na minha mente pois ao mesmo tempo que eu queria muito, não queria continuar trabalhando com receio de ser contaminada. Não queria falar com as pessoas o motivo do afastamento por estar_gestante pois estava bem no início. Falei que estava com sintomas de covid para não ir trabalho. Foi horrível pois ficavam cobrando o teste, quando iria retornar, a escala ficando apertada pois vários colegas se afastando por covid. No mesmo período as colegas do vínculo Fundação saúde foram remanejadas para outros hospitais. Depois quase perdemos 2 colegas e amigas do vínculo nerj ameaçaram as demitir. Foi um furacão nas nossas vidas que culminou com a perda do meu bebê não tinha batimentos. Agora bem melhor, me recuperando. Faço terapia particular online que foi o que me ajudou muito.

**** *ind_0172 *estr_2 *sex_2 *ida_2 *prof_9

Impotente frente às incertezas.

**** *ind_0173 *estr_2 *sex_2 *ida_1 *prof_4

Está sendo um desafio sendo recém formada e enfrentar uma situação extrema, mas acredito que serviu de experiência de como me comportar e reagir em situações_de_crise.

**** *ind_0176 *estr_2 *sex_2 *ida_1 *prof_3

Nos sentimos inseguros e com medo por nós e pelas pessoas do nosso convívio, um nível_de_ estresse alto por sobrecarga_de_trabalho e uma ansiedade que acabe logo tudo isso e voltemos as atividades_normais.

**** *ind_0177 *estr_2 *sex_2 *ida_1 *prof_5

Com o home_office acabamos trabalho mais que o habitual e se sentindo meio que na obrigação de trabalho o tempo todo. A pandemia gerou muita ansiedade e ter que ser produtivo apesar de tudo me gerou mais estresse e sensação de angústia.

**** *ind_0184 *estr_1 *sex_2 *ida_2 *prof_5

A pandemia me trouxe uma oportunidade de trabalho com tipo de paciente quenão estou acostumada. Associado a isso, existe o medo da contaminação, gerando um estresse redobrado. Atualmente, meencontro mais adaptada ao trabalho quefui designada a fazer, mas ainda tenho momentos de insegurança. Tenho certeza que este é um momento de aprendizado para todos.

**** *ind_0187 *estr_2 *sex_2 *ida_1 *prof_3

Me sinto isolada. As vezes saio na_rua por necessidade, e percebo que são poucas as pessoas que fazem por exemplo o uso_de_máscara que é algo tão simples e eficaz. Fico triste por ver as pessoas não levarem a sério o que está acontecendo no país e no mundo.

**** *ind_0192 *estr_2 *sex_2 *ida_2 *prof_7

Incertezas.

**** *ind_0204 *estr_2 *sex_2 *ida_2 *prof_7

Insegurança e falta de otimismo.

**** *ind_0207 *estr_2 *sex_2 *ida_3 *prof_3

Então, devido eu ser uma pessoa muito introvertida a pandemia me deixou ainda mais introspectiva. Observamos no início a dificuldade e o medo que as pessoas tinham umas das outrase até hoje algumas pessoas são bastante agressivas, se mesmo com a máscara para se protegerem observarem que o outro está sem esta proteção ficam irritados e destratam o outro.

**** *ind_0208 *estr_1 *sex_2 *ida_1 *prof_7

No momento, me sinto impotente por estar_gestante e com asma, gostaria de cooperar mais com meus colegas_de_trabalho, ao mesmo tempo que me sinto mais segura e tranquila por exercer o trabalho_remoto

**** *ind_0209 *estr_2 *sex_2 *ida_1 *prof_3

Me sinto insegura e como medo ,mesmo que já tenha tido a doença. Me sinto constantemente cansada.

**** *ind_0210 *estr_1 *sex_2 *ida_3 *prof_7

Esperançosa com a vacina. Saudade dos amigos, família e de passear.

**** *ind_0211 *estr_1 *sex_2 *ida_2 *prof_14

Como profissional de saúde me sinto desrespeitada por não ter acesso a epi devidamente. É tudo no improviso de péssima_qualidade.

**** *ind_0215 *estr_1 *sex_1 *ida_2 *prof_11

Me sinto um perfeito idiota com a quantidade de discursos absolutamente divergentes, inclusive entre os profissionais_da_área_de_saúde.

**** *ind_0228 *estr_1 *sex_2 *ida_2 *prof_7

Abertura total.

**** *ind_0233 *estr_2 *sex_2 *ida_1 *prof_3

Minha pressão_arterial aumentou. Sinto frequentemente a pulsação em minha cabeça.

**** *ind_0241 *estr_2 *sex_2 *ida_2 *prof_7

Muito cansada.

**** *ind_0242 *estr_1 *sex_2 *ida_2 *prof_7

Acho que o que mais incomoda, é não termos certeza de nada. Não ter prazo para terminar, não saber o que vai acontecer no momento seguinte.

**** *ind_0246 *estr_1 *sex_1 *ida_3 *prof_7

Me sinto por vezes deprimido por estar afastado de meu ambiente de trabalho na instituição por fazer parte de grupo_de_risco.

**** *ind_0249 *estr_1 *sex_1 *ida_3 *prof_7

Sinto me tranquilo desempenhando normalmente minhas funções com todos os cuidados de_prevenção.

**** *ind_0253 *estr_1 *sex_2 *ida_2 *prof_14

Bom trabalho a todos. Nesse momento estou confiante na vacina muito em breve e a vida voltar ao novo_normal. Obrigada.

**** *ind_0256 *estr_2 *sex_2 *ida_2 *prof_3

Não consigo concluir nenhum trabalho, não consigo finalizar nem mesmo a leitura de um livro.

**** *ind_0259 *estr_2 *sex_2 *ida_2 *prof_7

Me sinto sobrecarregada com o trabalho, afazeres_domésticos que não fazia antes pois minha ajudante está isolada, com minhas filhas e minha mãe_idosa que está muito deprimida, meu marido de home_office mal_humorado.

**** *ind_0260 *estr_1 *sex_2 *ida_3 *prof_3

Sou considerada idosa. Tive covid em abril e estou liberada do trabalho até a presente data. Sou bipolar e utilizo antipsicóticos.

**** *ind_0265 *estr_2 *sex_2 *ida_1 *prof_14

Desenvolvi sinusite 10 dias após diagnóstico de covid.

**** *ind_0269 *estr_1 *sex_2 *ida_1 *prof_7

Achei muito interessante e sugiro questões sobre família que tiveram o covid.

**** *ind_0270 *estr_1 *sex_2 *ida_2 *prof_3

Preocupada com meus pais.

**** *ind_0276 *estr_2 *sex_2 *ida_2 *prof_14

Eu me senti muito vulnerável com medo quando eu me contaminei senti uma sensação de morte mais graças a deus estou bem.

**** *ind_0280 *estr_1 *sex_2 *ida_3 *prof_3

Me sinto normal como antes da pandemia.

**** *ind_0282 *estr_1 *sex_2 *ida_2 *prof_11

Me sinto insegura diante dessa pandemia, principalmente quando você vê que tem pessoas que não usam máscaras e estão nas_ruas como se não estivesse acontecendo nada. Isso me irrita profundamente.

**** *ind_0288 *estr_2 *sex_2 *ida_2 *prof_1

O descaso de algumas pessoas com a pandemia me gera um sentimento de impotência enorme, um desânimo muito grande. O aspecto de minha_vida mais afetada durante a pandemia foi minha concentração não consigo estudar, ler, assistir televisão ou coisa do tipo.

**** *ind_0294 *estr_1 *sex_2 *ida_2 *prof_11

Indignada por ver pessoas na_rua sem máscara e frequentando lugares públicos como se não estivéssemos atravessando uma pandemia. Sinto que essas pessoas sentem que precisam muito se divertir e os otários é que ficam em_casa. Às vezes penso que tem alguém fazendo papel de idiota nessa história. não sei ainda se sou eu que estou em_casa ou eles que querem se divertir a qualquer preço. Falta empatia.

**** *ind_0297 *estr_2 *sex_1 *ida_2 *prof_7

Ansioso e deprimido com a situação do país.

**** *ind_0302 *estr_1 *sex_2 *ida_2 *prof_3

Como profissional, fiquei muito preocupada o que poderia acontecer até porque vi colegas ir ao óbito.

**** *ind_0303 *estr_1 *sex_2 *ida_2 *prof_3

Me sinto bem.

**** *ind_0305 *estr_1 *sex_2 *ida_2 *prof_7

O isolamento_social é o mais desagradável. O medo contrair a doença por não sabermos o que pode acontecer assusta bastante. No entanto, é preciso manter o bom_senso e a calma. Criar rotinas de proteção e não sair delas, para tentar não contrair a doença.

**** *ind_0307 *estr_2 *sex_2 *ida_2 *prof_7

Me sinto impotente e preocupada.

**** *ind_0310 *estr_1 *sex_2 *ida_3 *prof_7

Surpreendentemente me sinto muito bem. Aproveitei a pandemia para iniciar uma yoga, intensificar as meditações, fazer cursos, ler livros, fazer jejum intermitente. Transformei um período difícil em momento de autoconhecimento e crescimento_pessoal.

**** *ind_0314 *estr_1 *sex_2 *ida_2 *prof_3

O pior da pandemia é a ausência de políticas_do_governo para a combater.

**** *ind_0315 *estr_2 *sex_2 *ida_2 *prof_11

Tive alguma dúvida em relação a como me sentia no início da pandemia, sem epi, informações, sintomas que perduraram por semanas, mas após quase três meses desapareceram, por exemplo, atualmente os sentimentos e sintomas são outros.

**** *ind_0320 *estr_2 *sex_2 *ida_1 *prof_6

Exausta. Apesar de não lidar diretamente com o covid, atender o público_infantil e acho que o estresse geral acabou trazendo ainda mais estresse para nossa atuação, sem contar que os responsáveis em_geral achavam que por estarmos atendendo remotamente, estávamos à toa, sendo que eu tinha que planejar previamente uma terapia para cada um deles. Enfim, foi muito estresse o atendimento_remoto, ainda mais quando associamos com o estresse de ter que se expor novamente. Aí que o balde entornou.

**** *ind_0322 *estr_2 *sex_2 *ida_1 *prof_7

Apesar do meu estresse em meio a essa situação do covid, penso que minha religião e minha fé têm me suportado e sido minha âncora para que eu não pense que minha_vida está fora do controle.

**** *ind_0325 *estr_1 *sex_2 *ida_2 *prof_10

Ajuda mútua.

**** *ind_0326 *estr_1 *sex_2 *ida_2 *prof_7

Deprimida.

**** *ind_0327 *estr_1 *sex_2 *ida_2 *prof_3

Estressada, cansada, decepcionada com atitudes de pessoas que subestimam a doença.

**** *ind_0331 *estr_2 *sex_2 *ida_1 *prof_13

Entediada.

**** *ind_0332 *estr_2 *sex_2 *ida_1 *prof_14

Depois da pandemia a única coisa que posso dizer em poucas palavras é que não sou a mesma pessoa que antes em n situações da minha vida.

**** *ind_0333 *estr_2 *sex_1 *ida_1 *prof_3

Sugiro a colocação de questões relacionadas ao convívio família dos profissionais de saúde, expectativas e incertezas da profissão. Questões relacionadas ao envolvimento dos gestores com a saúde do profissional da ponta.

**** *ind_0334 *estr_1 *sex_2 *ida_2 *prof_7

Chateada.

**** *ind_0337 *estr_1 *sex_2 *ida_1 *prof_3

Me sinto desvalorizada como profissional da saúde, salários atrasados, sem receber o dissídio, o que devíamos receber por direito quanto nossa exposição. Sem treinamento, realizando procedimentos sem ser perito. Pois em relação ao covid, tudo é novo.

**** *ind_0342 *estr_2 *sex_2 *ida_1 *prof_1

Muito angustiada e ansiosa.

**** *ind_0343 *estr_1 *sex_2 *ida_2 *prof_3

Tenho dupla vinculação profissional. Uma delas na docência e outra na assistência. Sinto maior sobrecarga relacionada a dificuldades de gerenciar o tempo de transição entre a saída do hospital, os cuidados de higiene ao adentrar em_casa e iniciar a segunda etapa do dia, que diz respeito às demandas da instituição de ensino.

**** *ind_0344 *estr_1 *sex_2 *ida_2 *prof_7

Tristeza muito grande.

**** *ind_0349 *estr_2 *sex_2 *ida_2 *prof_7

No momento um breve alívio por mudanças de atividades saí temporariamente do monitoramento remoto de doentes para poder atender outras demandas. Tenho percebido que a mudança radical do cenário não foi o problema e, sim, o volume de demandas de atendimento e a tensão dos pacientes mesmo quando apresentavam quadros leves. Percebo que outras pessoas, mesmo de outras profissões e áreas de atuação, abordam o assunto distanciamento social querendo dizer que também estão passando por estresse tentando com isso abrir espaço para um suporte mútuo. Essa abordagem estamosjuntos tem sido reconfortante.

**** *ind_0350 *estr_2 *sex_2 *ida_2 *prof_7

Ao mesmo tempo que me sinto angustiada, tem sido uma oportunidade de conviver mais com a minha família e perceber questões que na minha rotina corrida de trabalho não via. Tenho está oportunidade porque a maior parte do meu trabalho tem ocorrido remotamente.

**** *ind_0352 *estr_2 *sex_2 *ida_1 *prof_1

Penso que os primeiros meses da pandemia foram piores para mim, estava em pânico, com medo sair para trabalho, pensava com frequência que algo ruim pudesse a acontecer, sentimentos e pensamentos sobre a morte. Isolamento_social e principalmente da família, meus pais e irmãos, pesaram muito nesse momento. O que me ajudou bastante foi o apoio psicológico. Atualmente, estou bem melhor, talvez mais adaptada à essa realidade. Além de ter visitado os meus pais, esporadicamente, neste último mês.

**** *ind_0353 *estr_1 *sex_1 *ida_2 *prof_9

Inseguro.

**** *ind_0354 *estr_1 *sex_2 *ida_3 *prof_9

Cansada.

**** *ind_0356 *estr_2 *sex_2 *ida_1 *prof_4

Estressada.

**** *ind_0357 *estr_1 *sex_2 *ida_1 *prof_7

Muito_tranquila.

**** *ind_0359 *estr_2 *sex_2 *ida_2 *prof_1

A palavra que define hoje meu momento é medo.

**** *ind_0360 *estr_1 *sex_1 *ida_1 *prof_5

Tenho minha consciência muito_tranquila em_relação a mim, mas me preocupa o fato dos outros não estarem fazendo mais do que poderiam.

**** *ind_0363 *estr_2 *sex_2 *ida_2 *prof_14

Exausta, triste e com muito medo não de adoecer, mas principalmente de contaminar meus filhos que são muito alérgicos, pânico de os perder.

**** *ind_0373 *estr_2 *sex_2 *ida_1 *prof_14

Cansada.

**** *ind_0376 *estr_1 *sex_2 *ida_2 *prof_14

Me sinto sempre com a expectativa de que essa pandemia vai se prolongar por muito tempo.

**** *ind_0378 *estr_1 *sex_2 *ida_3 *prof_7

Me sinto muito bem por ter tido uma forma quase assintomática de covid e por continuar a ser útil aos pacientes no trabalho diário.

**** *ind_0379 *estr_1 *sex_2 *ida_2 *prof_7

Tranquilo.

**** *ind_0381 *estr_2 *sex_2 *ida_2 *prof_7

No momento um breve alívio por mudanças de atividades saí temporariamente do monitoramento remoto de doentes para poder atender outras demandas. Tenho percebido que a mudança radical do cenário não foi o problema e, sim, o volume de demandas de atendimento e a tensão dos pacientes mesmo quando apresentavam quadros leves. Percebo que outras pessoas, mesmo de outras profissões e áreas de atuação, abordam o assunto distanciamento_social querendo dizer que também estão passando por estresse tentando com isso abrir espaço para um suporte mútuo. Essa abordagemestamos juntos tem sido reconfortante.

**** *ind_0384 *estr_2 *sex_2 *ida_1 *prof_3

Estou irritada por estar longe da minha família. Sensação de não estar aguentando mais. Tenho comido muito e estou com muitos problemas para dormir.

**** *ind_0387 *estr_1 *sex_2 *ida_2 *prof_11

Sinto a vulnerabilidade da vida de forma mais clara e presente o que me inquieta.

**** *ind_0388 *estr_1 *sex_2 *ida_2 *prof_7

Me sinto privilegiada por ter tido a oportunidade_de_trabalho em home_office pela segurança de manter o isolamento_social.

**** *ind_0392 *estr_2 *sex_2 *ida_1 *prof_1

Me sinto importante, pois que pandemia é algo que foge ao meu controle. Porém mantenho a mente firme, com expectativas de que em um futuro próximo, dias melhores virão.

**** *ind_0405 *estr_1 *sex_2 *ida_3 *prof_3

Esse é um momento de exercício de paciência, já foi mais estresse, com o isolamento_social total, mais agora poder voltar ao trabalho é muito_bom e aproveitar o que o mundo_virtual, pode nos proporcionar de_bom, mas nada muda a nossa liberdade de ir e vir.

**** *ind_0408 *estr_1 *sex_1 *ida_3 *prof_7

Como fiquei afastado do atendimento durante o período, em quarentena, não tive problemas financeiros, não me senti muito afetado.

**** *ind_0410 *estr_2 *sex_2 *ida_1 *prof_4

O maior estresse é a falta de protocolo mundial. Cada médico prescreve como acha melhor. Dentro de uma Instituição Pública fica difícil padronizar por causa da alta rotatividade desses profissionais, principalmente em emergência.

**** *ind_0413 *estr_2 *sex_2 *ida_2 *prof_7

Esgotada mentalmente devido_a inúmeras_informações.

**** *ind_0414 *estr_2 *sex_2 *ida_2 *prof_14

Me sinto muito angustiada. Perdi amigos, conhecidos e outros que todos os dias, perecem da doença. Apesar de não trabalho diretamente com pacientes com covid, vira e mexe aparece um contaminado, daí tem que testar todo mundo, transfere os que positivaram para a enfermaria específica, fecha o setor para desinfeção depois volta tudo de novo. O medo pegar ou passar para família é desesperador. Ainda por cima temos que lidar com pessoas que até hoje, depois de tantas mortes, acham que é tudo exagero da mídia, que é contra o isolamento_social, não segue as normas colocando em risco a própria vida e dos outros. Também tem as informações desencontradas que deixa a população_perdida, sem saber o que é real e o que é fake. Governantes agindo de má intenção. Muito triste essa situação.

**** *ind_0418 *estr_1 *sex_2 *ida_2 *prof_9

Sinto que não temos controle sobre as circunstâncias, mas as nossas reações a elas são termômetro para nos mostrar quem somos e o que podemos mudar em nós, através de um novo modo de enxergar a vida. Que somos impotentes e a pandemia faz parte dessa reflexão. Que o momento nos igual a, o que nos permite várias reflexões. Sair do eu parao nós, nunca foi tão necessário. Que o momento é um grande despertar.

**** *ind_0421 *estr_2 *sex_2 *ida_2 *prof_11

Insegura.

**** *ind_0423 *estr_1 *sex_2 *ida_3 *prof_7

Homeopatia é uma grande ajuda tanto na prevenção quanto o tratamento do covid.

**** *ind_0424 *estr_1 *sex_1 *ida_2 *prof_7

Me sinto tranquilo no momento. Estive bastante cansado e desgastado no auge da pandemia no meu Estado.

**** *ind_0433 *estr_1 *sex_2 *ida_2 *prof_5

Me sinto solidária e extremamente perplexa em relação a falta de salário de muitos profissionais_de_saúde que estiveram e estão na linha de frente. E extremamente decepcionada com a política_nacional.

**** *ind_0436 *estr_2 *sex_2 *ida_2 *prof_5

Fico triste de ver o povo abusando ou não acreditando na seriedade da pandemia. Meu pai faz parte das estatísticas infelizmente, e sei, na própria pele o que a covid pode fazer.

Acho que ainda é cedo para a liberação do comércio pois a incidência de mortos está muito alta.

**** *ind_0440 *estr_1 *sex_2 *ida_1 *prof_3

As vezes cansada devido_a muito_trabalho.

**** *ind_0442 *estr_2 *sex_2 *ida_2 *prof_8

Me sinto muito ansiosa por ser do grupo de risco. Minha_mãe tem 82_anos e alzheimer, minha_filha tem asma e meu_marido é fumante e tem 65_anos. E somente eu tenho que sair para trabalho. Qualquer tosse de alguém da_família fico apavorada.

**** *ind_0443 *estr_1 *sex_2 *ida_2 *prof_7

A incerteza do amanhã é preocupante, mas sei que vai passar. Me cuidado e espero que novos bons hábitos sejam incorporados à nossas vidas.

**** *ind_0445 *estr_2 *sex_2 *ida_2 *prof_7

Com medo.

**** *ind_0447 *estr_1 *sex_2 *ida_2 *prof_14

Com todo cuidado que a situação da covid exige, comigo mesma, família e pacientes, o ministério_da_saúde demitiu a mim e aos meus colegas. Eu tinha esse trabalho. O contrato não contempla rescisão. O nervosismo pelo desemprego, contribui ainda mais ao estresse.

**** *ind_0449 *estr_2 *sex_2 *ida_2 *prof_3

Com medo retornar ao trabalho e haver nova contaminação e triste por não ter no momento o prazer de trabalho que sentia. Decepcionada por ver que ninguém na instituição se preocupa com você ou te oferece alguma ajuda após uma vida toda de trabalho.

**** *ind_0450 *estr_1 *sex_2 *ida_2 *prof_11

Me sinto com medo e confiante.

**** *ind_0452 *estr_2 *sex_2 *ida_2 *prof_3

Consumida.

**** *ind_0453 *estr_2 *sex_2 *ida_2 *prof_7

Está pandemia veio confirmar que nada está sob controle na vida. Que devemos apoiar uns aos outros e proteger quem amamos. Está catástrofe poderia ter sido de fato evitada se medidas responsáveis tivessem sido tomadas por organizações mundiais de controle.

**** *ind_0454 *estr_1 *sex_2 *ida_3 *prof_3

Tranquila. Religião espírita dá suporte e compreensão deste momento.

**** *ind_0456 *estr_1 *sex_2 *ida_2 *prof_9

A pandemia é diferente para diferentes pessoas. Moro numa casa_bacana, tenho trabalho, consigo isolamento_social, mas trabalho por ser profissional de saúde. Sigo as normas da oms com relação ao uso_de_máscara e distanciamento_social. Enfim, para mim está sendo razoavelmente tranquilo. Triste pela perda de alguns conhecidos. Assustada às vezes. Abismada com tantos_mortos.

**** *ind_0458 *estr_2 *sex_2 *ida_2 *prof_7

Estável.

**** *ind_0462 *estr_2 *sex_2 *ida_1 *prof_7

Cansada, esgotada física e mentalmente, com medo contrair covid e passar aos meus família e algum deles vir a óbito.

**** *ind_0465 *estr_2 *sex_2 *ida_1 *prof_3

Foi período de grande estresse e desafios. Mas também de superação e crescimento profissional em um período curto de tempo.

**** *ind_0467 *estr_1 *sex_2 *ida_2 *prof_7

Medo adoecer e não saber a evolução. Medo contaminar idosos como meus pais. Medo do caos escolar e econômico.

**** *ind_0469 *estr_2 *sex_2 *ida_1 *prof_3

Me sinto desestimulada, cansada mentalmente e fisicamente. É desgastante lidar com mais esse problema e a falta de empatia do outro para comigo.

**** *ind_0471 *estr_2 *sex_2 *ida_2 *prof_3

Triste vendo tantas mortes, mesmo não estando diretamente na assistência.Muito impactado de como a vida mudou da água parao vinho em tão poucos meses, mas o isolamento_social da família e a sobrecarga_de_trabalho foram o que mais me afetaram.

**** *ind_0474 *estr_1 *sex_2 *ida_2 *prof_14

Choro muito sozinhaescondido tenho perdido muitos conhecidos da saúde por covid e quando vejo as pessoas sem seguir as orientações sem_máscara desfilando na_rua e escuto pessoas internadas contando daquele família que não respeitou ninguém dentro de_casa e acabou contaminando quem estava se cuidado.

**** *ind_0476 *estr_1 *sex_2 *ida_2 *prof_7

Uma sensação de fraqueza, submissão a uma realidade muito diferente.

**** *ind_0479 *estr_1 *sex_2 *ida_1 *prof_3

Sinto muita falta de abraçar e beijar, meus colegas_de_trabalho e pacientes. Esta doença nos tem tirado a possibilidade de sermos mais solidários aos nossos pacientes através de pequenos gestos como um abraço.

**** *ind_0483 *estr_2 *sex_2 *ida_2 *prof_7

Foi muito difícil passar por esse período sozinha, com dois filhos, trabalho muito, com ajuda apenas da minha babá, longe dos meus pais, irmãos e amigos, tendo que administrar o home_office entre outras coisas.

**** *ind_0484 *estr_1 *sex_2 *ida_3 *prof_7

Nesse momento, as coisas estãomelhores, devido_a diminuição dos casos_de covid menos estresse, mais confiante, devido, sentir mais segura em evitar contaminação.

**** *ind_0490 *estr_1 *sex_1 *ida_2 *prof_7

Sinto cansado e esperando a normalização.

**** *ind_0498 *estr_1 *sex_2 *ida_2 *prof_11

Me sinto bem. Contribuiu positivamente em muitos aspectos.

**** *ind_0499 *estr_1 *sex_2 *ida_1 *prof_8

Me sinto triste com a falta de perspectiva de melhora.

**** *ind_0505 *estr_1 *sex_2 *ida_1 *prof_7

Nesse momento que o número_de_casos diminuiu bastante, me sinto mais_tranquila, confiante que a situação está se controlando aos poucos.

**** *ind_0506 *estr_1 *sex_2 *ida_3 *prof_11

Por eu estar ainda, nessemomento, com a presença de anticorpos de fase aguda, estou apreensiva em relação aos sintomas. Se podem se agravar.

**** *ind_0508 *estr_2 *sex_2 *ida_2 *prof_7

Me sinto ainda triste, mas esperançosa.

**** *ind_0513 *estr_1 *sex_2 *ida_2 *prof_7

A fé é crer em deus foi o ponto mais forte para sair do cti.

**** *ind_0515 *estr_1 *sex_1 *ida_2 *prof_11

Tranquilo e prevenido. Cumpro todos os protocolos.

**** *ind_0517 *estr_2 *sex_2 *ida_1 *prof_14

Pânico.

**** *ind_0525 *estr_2 *sex_2 *ida_1 *prof_15

Cheia de desafios a ser vencido e como pequena empresária triste em não ter um apoio real do governo.

**** *ind_0529 *estr_1 *sex_2 *ida_2 *prof_3

Assustada.

**** *ind_0535 *estr_2 *sex_2 *ida_1 *prof_11

Me sinto exausta. Extremamente cansada e sem perspectiva de que sairemos melhores. A ausência de uma política_pública articulada em os três poderes contribui para que haja uma sensação de que não iremos sair disto nunca.

**** *ind_0536 *estr_2 *sex_2 *ida_2 *prof_7

Tristeza.

**** *ind_0537 *estr_2 *sex_2 *ida_2 *prof_7

Sinto que deus está no controle de tudo e me esforço ao máximo para fazer a minha parte no processo usando todos os epi.

**** *ind_0539 *estr_2 *sex_1 *ida_1 *prof_7

Sinto culpa por não ter trabalhado o suficiente e por ter saído da linha_de_frente antes da explosão verdadeira, apesar de entender que não tinha condições de trabalho naquelas circunstâncias.

**** *ind_0541 *estr_1 *sex_2 *ida_2 *prof_5

Sinto feliz por ter ajudado salvar algumas vidas e extrema frustração da falta de respeito dos governantes não pagarem nossos salários. Triste realidade.

**** *ind_0545 *estr_2 *sex_1 *ida_1 *prof_1

Eu tenho sentindo muito cansada, angustiada e preocupada com o futuro, pois eu tenho me cuidado e pensando nos demais. Mas as pessoas têm feito totalmente ao contrário.

**** *ind_0547 *estr_1 *sex_2 *ida_1 *prof_3

Gostaria que contemplasse as pessoas que tiveram sinais e sintomas de covid e que os exames não foram diagnosticados.

**** *ind_0548 *estr_2 *sex_2 *ida_2 *prof_7

Me sinto incapaz de melhorar as deficiências que observo na saúde. Na falta de respeito com os profissionais quer no setor_público ou no setor_privado. A valorização não é real agora, nem antes, e não vejo possibilidades de mudança.

**** *ind_0550 *estr_2 *sex_2 *ida_2 *prof_14

Desanimada.

**** *ind_0552 *estr_1 *sex_2 *ida_2 *prof_14

Sinto medo me contaminar é levar para minha família. Sinto que nós profissionais_de_saúde damos nossa vida pelos nossos pacientes.

**** *ind_0556 *estr_1 *sex_2 *ida_1 *prof_3

Péssima qualidade de vida.

**** *ind_557 *estr_2 *sex_2 *ida_2 *prof_7

Largada, desamparada, desrespeitada, exausta, deprimida, sem esperança, desanimada, endividada, fiquei 2 meses sem receber minha principal fonte, humilhada.

**** *ind_0558 *estr_2 *sex_1 *ida_1 *prof_7

Aliviado por já ter tido a doença sem nenhum agravo.

**** *ind_0559 *estr_1 *sex_2 *ida_3 *prof_8

Apesar de ter ficado mais sobrecarregada com os afazeres domésticos e continuando trabalho no hospital, me considero felizarda por não ter sido atingida na quarentena. Até porque estou num período tranquilo de minha vida, não me afetou financeiramente e tenho uma força de fé muito grande. fiquei mais ansiosa pela limitação de movimento fora de_casa.

**** *ind_0562 *estr_1 *sex_2 *ida_2 *prof_11

Adaptada e com um pouco de dificuldade de planejamento do retorno à dinâmica normal.

**** *ind_0564 *estr_2 *sex_2 *ida_1 *prof_14

Estou extremamente estressada preciso de ajuda_psicológica.

**** *ind_0565 *estr_1 *sex_1 *ida_2 *prof_3

É necessário disponibilizar testes rápidos de qualidade para profissionais_de_saúde e apoio no_geral.

**** *ind_0567 *estr_1 *sex_2 *ida_3 *prof_6

Me sinto receosa pois as pessoas estão afrouxando nos cuidados.

**** *ind_0568 *estr_2 *sex_2 *ida_2 *prof_7

Ansiedade bastante aumentada pelas incertezas e pelo controle inadequado dos governantes e da população.

**** *ind_0569 *estr_1 *sex_2 *ida_2 *prof_14

Neste exato momento como profissional da área_de_saúde me sinto impotente diante de uma doença tão grave, ver tanto descaso de autoridades que se demonstram inerte a tantas situações e não olham para quem de fato necessita de um olhar diferenciado e atenção agindo com descaso com tantas desinformação sem contar com tanta corrupção deixando que assim tantas pessoas fossem a óbitos sem a menor chance de tratamento.

**** *ind_0571 *estr_2 *sex_2 *ida_2 *prof_3

Impotente.

**** *ind_0572 *estr_1 *sex_1 *ida_1 *prof_10

Estou me sentindo muito bem. A pandemia me ajudou a repensar algumas questões e avançar em projetos que estavam quase engavetados.

**** *ind_576 *estr_1 *sex_2 *ida_2 *prof_7

O sentimento maior é de ansiedade quando será possível voltar próximo ao antigo normal, quando sentiremos quehá segurança. Uma certa solidão pela falta de convivência cm os amigos. Sentimento profundo de satisfação de ver tantas empresas doando e ajudando e por outro lado a vergonha da Intensa corrupção quese viu por parte dos governates mostrando mais ainda a ineficiência do estado. Falta tudo no meu hospital que é federal mas virou referência covid faltam exames laboratoriais simples, monitores, atendimento não covid.

**** *ind_0578 *estr_1 *sex_2 *ida_3 *prof_11

Preocupada com o desgoverno que o país está passando e as inúmeras mortes que poderiam ter sido evitadas.

**** *ind_0581 *estr_2 *sex_2 *ida_2 *prof_3

Infeliz.

**** *ind_0584 *estr_1 *sex_2 *ida_3 *prof_7

Impotente. Triste. Política de saúde ineficaz e corrupta a nível_federal, nível_estadual e nível_municipal. Negacionismo, negligência com à população.

**** *ind_0585 *estr_2 *sex_2 *ida_2 *prof_14

Como profissional_da_saúde, me sinto cansada e desmotivada a prosseguir na minha profissão.

**** *ind_0586 *estr_2 *sex_2 *ida_1 *prof_3

Me sinto sobrecarregado, descriminado e afastado do convívio com colegas devido_a minha_profissão e desrespeitada profissionalmente, lideranças são péssimos gestores.

**** *ind_0587 *estr_1 *sex_2 *ida_2 *prof_14

Me sinto cansada.

**** *ind_0588 *estr_2 *sex_2 *ida_1 *prof_3

Me senti insegura em trabalho e mesmo com todos os epi fornecidos de forma adequada pelo hospital, tive e tenho medo trazer contaminação a minha família. Tive ansiedade e sofri muito por não dar conta de tudo nesse período. Está sendo exaustivo e doloroso estar longe de todos, termos de nos privar desse contato para preservar a quem amamos.

**** *ind_0591 *estr_2 *sex_2 *ida_1 *prof_14

Presa, isolada, trabalho direto com pessoas com covid. Tenho medo pegar de novo. Fiquei sem chão não pude ficar com meus bebês sem contar que sofri descriminação.

**** *ind_0592 *estr_2 *sex_2 *ida_2 *prof_3

Me sinto angustiada.

**** *ind_0598 *estr_2 *sex_2 *ida_1 *prof_3

Me sinto sobrecarregado, descriminado e afastado do convívio com colegas devido_a minha_profissão e desrespeitada profissionalmente, lideranças são péssimos_gestores.

**** *ind_0602 *estr_1 *sex_2 *ida_1 *prof_7

Insegurança financeira.

**** *ind_0607 *estr_2 *sex_2 *ida_2 *prof_3

Poderiam perguntar sobre o medo_da_morte efetivamente e também sobre o medo não ter atendimento adequado no sistema_de_saúde.

**** *ind_0609 *estr_2 *sex_2 *ida_2 *prof_3

Temerosa.

**** *ind_0614 *estr_2 *sex_2 *ida_1 *prof_14

Estressada, cansada, vivo em constante ansiedade.

**** *ind_0618 *estr_1 *sex_2 *ida_2 *prof_3

Me sinto insegura, aborrecida por não poder estar livre em contato com família e por não saber quando tudo isso vai terminar.

**** *ind_0619 *estr_2 *sex_2 *ida_2 *prof_7

Exausta.

**** *ind_0622 *estr_2 *sex_2 *ida_1 *prof_3

Me sinto vulnerável, sem ajuda, tanto da instituição a qual trabalho quanto psicológica

**** *ind_0623 *estr_1 *sex_2 *ida_1 *prof_5

Com saudades do convívio família.

**** *ind_0624 *estr_1 *sex_2 *ida_1 *prof_7

Me sinto mais certa do controle de deus sobre todas as coisas do que nunca.

**** *ind_0626 *estr_1 *sex_2 *ida_1 *prof_8

Momento especial de autoconhecimento, incluindo limitações que fugiram da minha vontade.

**** *ind_0629 *estr_2 *sex_2 *ida_1 *prof_5

Ansiosa.

**** *ind_0630 *estr_1 *sex_2 *ida_2 *prof_8

Apreensiva, mas confiante na superação.

**** *ind_0633 *estr_2 *sex_1 *ida_1 *prof_7

Me sinto impotente. Meu pai, de 52_anos, HAS controlado, apenas, faleceu de covid. Minha vida mudou depois disso. Minha_mãe e eu não apresentamos sintomas. Eu sou igg, ela não. Tenho medo ainda contaminá-la e ela ficar grave. Como pode eu salvar tantas vidas pelo covid e perder uma das vidas mais importantes que estava ao meu lado. Eu o tratei do jeito certo, mas não foi o suficiente. Não me sinto_mal, achando que deveria ter feito mais medicações, mas me sinto mal por ele ter morrido sendo que eu já salvei tantos outros com risco maiores que o dele.

**** *ind_0641 *estr_1 *sex_2 *ida_2 *prof_5

Meu desconforto é não poder abraçar meus pacientes. Minha família está comigo e estamos bem, então tudo bem. A dúvida do tempo que a pandemia vai levar é um fator de estresse importante.

**** *ind_0650 *estr_1 *sex_2 *ida_1 *prof_14

Me sinto estressada, chateada e amedrontada.

**** *ind_0651 *estr_2 *sex_2 *ida_1 *prof_14

A vida não é mais a mesma, falta algo.

**** *ind_0653 *estr_1 *sex_2 *ida_1 *prof_8

Me sinto bem, prático meditação. Também, o entendimento sobre espiritualidade tem me ajudado bastante nesse momento.

**** *ind_0657 *estr_1 *sex_2 *ida_2 *prof_5

Me sinto sem objetivo futuro.

**** *ind_0658 *estr_1 *sex_1 *ida_1 *prof_7

Todos nós passamos por diversas situações diariamente, preocupações, medos, certezas e incertezas, momentos que estamos confiantes e outros não. A diferença está em sermos líquidos capazes de nos adaptar ao ambiente situação onde nos encontramos. Parabéns pela iniciativa.

**** *ind_0660 *estr_1 *sex_2 *ida_1 *prof_3

Me sinto exposta a todo instante, sinto que a sociedade não está levando a sério a doença, em contrapartida os casos aumentando em minha cidade, a enfermagem tão importante nesse processo, linha_de_frente, estamos perdendo muito dos nossos para esse vírus e a desvalorização permanece. Tanto salarial quanto o respeito que merecemos por sermos peças fundamentais nesse jogo.

**** *ind_0661 *estr_2 *sex_2 *ida_2 *prof_7

Piora da depressão.

**** *ind_0663 *estr_1 *sex_2 *ida_3 *prof_8

Me sinto bem_tranquila, realizando todas as medidas_de_segurança tanto no serviço quanto em_casa ou quando tenho que ir à rua. Não vejo motivo para pânico.Com relação a sugestões: Acho que deveria ser abordado se no seu local_de_trabalho, desde o início da pandemia, foi oferecido algum tipo de epi para realizar o serviço com segurança.

**** *ind_0667 *estr_1 *sex_1 *ida_2 *prof_10

Me sinto muito estressado com o quadro geral da pandemia e o total descontrole por parte dos governantes e falta de consciência coletiva da sociedade.

**** *ind_0668 *estr_1 *sex_2 *ida_3 *prof_11

Triste pelas mortes e preocupada.

**** *ind_0669 *estr_2 *sex_2 *ida_2 *prof_5

Sinto medo contaminação, por ter uma mãe_idosa.

**** *ind_0671 *estr_1 *sex_2 *ida_2 *prof_7

Me sinto insegura, com medo contaminar a mim e à minha família. Falta capacitação par a lidar com a doença em todos os aspectos. Falta apoio da Instituição onde trabalho. Trabalho num local insalubre.

**** *ind_0672 *estr_1 *sex_2 *ida_1 *prof_5

Tranquila.

**** *ind_0675 *estr_2 *sex_2 *ida_2 *prof_11

Cansada e frustrada.

**** *ind_0680 *estr_1 *sex_2 *ida_2 *prof_5

Frequentemente tensa e angustiada.

**** *ind_0681 *estr_2 *sex_2 *ida_2 *prof_5

As relações de forma geral irão se estreitar ou abrir de vez.

**** *ind_0686 *estr_2 *sex_2 *ida_1 *prof_5

Preocupada.

**** *ind_0688 *estr_2 *sex_2 *ida_2 *prof_14

Sei que me afetou muito essa pandemia, sinto muita tristeza, sinto vontade de chorar toda hora, antes quando perdíamos paciente era diferente, eu conseguia ser forte, ter auto controle e superar, durante a pandemia as coisas mudaram eu sofro muito pelas perdas, fico muito mal, me sentindo incompetente, como se a culpa fosse nossa, dos profissionais_de_saúde que não foram capazes de salvar eles, nunca me sentir assim.

**** *ind_0689 *estr_1 *sex_2 *ida_3 *prof_7

Isolada, mas conseguindo me ocupar, com os trabalhos online. Difícil ficar afastada de filhos e netos, e principalmente preocupada em relação a eles. E irritada com o governo_federal que não leva a sério a gravidade da pandemia. Acho que é isto que me causa irritabilidade, a irresponsabilidade do governo_federal.

**** *ind_0691 *estr_2 *sex_2 *ida_1 *prof_3

Sinto me fadigada e insatisfeita com a situação mundial, pior ainda quando instituições usam de política para prejudicar o povo e os deixar mais assustados.

**** *ind_0694 *estr_2 *sex_2 *ida_1 *prof_7

Extremamente exausta e pouco valorizada por nossos superiores. Infelizmente, não há qualquer direito para nós médicos, deveres e, frequentemente, nem mesmo a remuneração programada. Isso é frequente e desanimador. Piora a nossa saúde mental já deteriorada pelo dia a dia de trabalho.

**** *ind_0697 *estr_2 *sex_2 *ida_2 *prof_7

Desenvolvi depressão e toc após ter tido covid, estando em tratamento com psiquiatra e psicológico.

**** *ind_0700 *estr_1 *sex_1 *ida_1 *prof_14

Tranquilo, pois sei que esse vírus será combatido.

**** *ind_0702 *estr_1 *sex_2 *ida_3 *prof_7

Bastante preocupada principalmente porque a população não obedece ao isolamento_social.

**** *ind_0704 *estr_1 *sex_2 *ida_2 *prof_14

Quero minha_vida normal.

**** *ind_0710 *estr_2 *sex_2 *ida_1 *prof_3

Meu nível_de_estresse piorou bastante e afetou meu relacionamento afetivo, que infelizmente chegou ao fim devido_ao meu descontrole_emocional. Gostaria muito de fazer terapia para melhorar meu quadro psicológico.

**** *ind_0713 *estr_2 *sex_2 *ida_2 *prof_7

Nesse momento estou muito cansada e desanimada.

**** *ind_0714 *estr_1 *sex_2 *ida_3 *prof_11

Preocupada com minha saúde e de todos da família e cuidado para que tudo fique bem.

**** *ind_0719 *estr_1 *sex_1 *ida_1 *prof_3

Nós profissionais_da_saúde sentimos medo e angústia com a covid e de pegar e levar para_casa e contaminarmos nossas família.

**** *ind_0721 *estr_2 *sex_2 *ida_2 *prof_7

Vivo momentos de ansiedade alternando com outros de esperança, assim como de impotência com sensação de dever cumprido. Muitas incertezas ainda.

**** *ind_0722 *estr_2 *sex_2 *ida_2 *prof_4

Muito tarde para fazer essas perguntas. O pior no Rio já passou e também a horrívelsensação de medo, insegurança, ser desrespeitada por decisões verticais absurdas.

**** *ind_0731 *estr_2 *sex_2 *ida_2 *prof_3

Poderiam perguntar sobre o medo da morte efetivamente e também sobre o medo não ter atendimento adequado no sistema de saúde.

**** *ind_0733 *estr_1 *sex_2 *ida_2 *prof_7

Estou mais_tranquila frente a nova realidade de vida, do trabalho com uso_de_epi que limitam a respiração e as mudanças do novo_normal. Acredito que o mundo mudou e precisamos estar preparados para essas mudanças. Vamos sentir impacto econômico, mas mesmo assim otimista. Coisas novas e boas estão por acontecer.

**** *ind_0735 *estr_2 *sex_2 *ida_2 *prof_7

Tolhida de minhas liberdades individuais.

**** *ind_0736 *estr_2 *sex_2 *ida_2 *prof_7

Medo, muito medo. Pesadelo sem fim.

**** *ind_0737 *estr_2 *sex_2 *ida_2 *prof_7

Estressada.

**** *ind_0738 *estr_2 *sex_2 *ida_2 *prof_1

Preocupada, intolerante e trabalho para que o cidadão que atendo seja tratado com respeito.

**** *ind_0742 *estr_1 *sex_2 *ida_2 *prof_7

Perplexidade.

**** *ind_0744 *estr_2 *sex_2 *ida_2 *prof_3

O profissional de saúde esteve confinado em ir de um hospital para o outro como se todos os dias fossem segunda_feira.

**** *ind_0745 *estr_2 *sex_2 *ida_2 *prof_14

Me sinto como se estivéssemos sozinhos tendo que lutar com um vírus por nossa conta e risco, num país onde os governantes não estão nem um pouco preocupados. Ainda bem que nós temos a fiocruz que está nessa batalha incansável por vacina eficaz.

**** *ind_0747 *estr_1 *sex_2 *ida_2 *prof_7

Pior da pandemia afastamento dos idosos da família, cansaço_físico do trabalho, falta de gestão_política_da_crise, sentimento de estar à deriva, preocupação com o estado mental dos família

**** *ind_0749 *estr_2 *sex_1 *ida_2 *prof_7

Preocupado com a possibilidade de adoecer e ser internado solitariamente e de vir a morrer. Tenho doença crônica, mas optei por não me ausentar neste momento que a população mais precisa de atendimento na área_da_saúde, mas atendo tenso, preocupado em usar de forma errada o epi. É um tempo muito difícil. Gosto, como pediatra, de brincar com as crianças e neste momento está difícil para brincadeiras.

**** *ind_0754 *estr_1 *sex_2 *ida_2 *prof_7

Me sinto esperançosa. Acreditando que tudo isso vai passar. Acho que faltaram perguntas a respeito de contato cimos família do paciente com covid. Sobre o sentimento em relação a poder ajudar na pandemia de alguma forma. Ou seja, ser útil nesse momento apesar do medo adoecer. Confesso que ser útil nesse momento e me sentir ajudando outras pessoas e colegas me fez sentir melhor em relação a tudo isso.

**** *ind_0756 *estr_1 *sex_2 *ida_2 *prof_7

Triste.

**** *ind_0761 *estr_2 *sex_2 *ida_2 *prof_7

Meu filho tem 4_anos e está muito difícil o convívio neste momento sem escola pois ele fica muito irritado e agitado pela minha ausência frequente e também quando eu retorno, ele demonstra a raiva de ter sentido minha ausência. Então acaba que o convívio em_casa está muito difícil. O marido está trabalho muito remotamente e também se estressa e não dá a atenção devida ao filho o que gera ainda mais estresse. Na parte profissional, houve muita sobrecarga por conta da ausência de muitos profissionais_de_saúde afastados e também, creio eu, pelo medo inerente ao covid, levando a muitas reclamações dos profissionais_de_saúde e estresse para resolução dos problemas tentando atender a todos minimamente.

**** *ind_0762 *estr_1 *sex_2 *ida_1 *prof_12

Minhas respostas nessa EPS geralmente estão acompanhadas do seguinte pensamento: mas tudo bem ser assim. O que sinto bastante falta é de conversar sobre a vida com mais profundidade, e me deixar levar por devaneios existenciais. Algo que até pode acontecer virtual, mas não tem o mesmo sabor ao ocorrer pessoalmente.

**** *ind_0764 *estr_1 *sex_2 *ida_2 *prof_7

Decepção com comportamento de muitas pessoas de convívio regular.

**** *ind_0767 *estr_1 *sex_2 *ida_2 *prof_3

Impotência.

**** *ind_0769 *estr_1 *sex_2 *ida_1 *prof_7

Minha ansiedade em relação a um possível contágio e evolução para um quadro clínico grave, reduziu a partir do momento que tive um quadro brando da covid.

**** *ind_0770 *estr_2 *sex_2 *ida_2 *prof_7

Me sinto insegura por não saber como as coisas vão evoluir, e sinto falta do convívio_social. Me sinto insegura em relação a sociabilidade dos meus filhos.

**** *ind_0775 *estr_1 *sex_2 *ida_3 *prof_7

Respiro um pouco mais aliviada em relação ao início da pandemia.

**** *ind_0776 *estr_1 *sex_2 *ida_3 *prof_11

Com sentimento de dever cumprido.

**** *ind_0778 *estr_2 *sex_2 *ida_2 *prof_14

Apavorada, sem esperança, muito cansada.

**** *ind_0779 *estr_1 *sex_1 *ida_2 *prof_7

Uso dos testes rápidos para todos da área de saúde em contato com o covid.

**** *ind_0784 *estr_2 *sex_2 *ida_2 *prof_7

Neste momento um pouco mais_tranquila devido_a redução do número_de_casos, da_transmissão e da demanda no trabalho.

**** *ind_0788 *estr_2 *sex_2 *ida_2 *prof_7

Solitária.

**** *ind_0790 *estr_1 *sex_2 *ida_3 *prof_7

Não me sinto muito isolada porque contínuo em contato com os colegas e acho o meu trabalho importante.

**** *ind_0792 *estr_1 *sex_1 *ida_2 *prof_7

Sugestão, qual o percentual de redução ou aumento do seu volume de trabalho profissional, durante a Pandemia. O quanto isso te estressou.

**** *ind_0795 *estr_2 *sex_2 *ida_2 *prof_3

Me sinto impotente com as situações geradas pela pandemia, frustrada com os governantes e as políticas_de_saúde.

**** *ind_0803 *estr_2 *sex_2 *ida_2 *prof_7

Temerosa, mas com esperanças de um mundo melhor.

**** *ind_0804 *estr_1 *sex_2 *ida_2 *prof_7

Cansada.

**** *ind_0805 *estr_1 *sex_2 *ida_2 *prof_7

Eu trabalho no serviço público onde não preocupação com os epi e na instituição_privada estou em home_office com assistência total.

**** *ind_0806 *estr_2 *sex_2 *ida_1 *prof_5

Acho que teríamos que ter um auxílio_financeiro. Porque não conseguimos o atual auxílio_emergencial e muitos como eu, estão com dificuldade_financeira.

**** *ind_0808 *estr_1 *sex_2 *ida_2 *prof_7

Perplexidade.

**** *ind_0809 *estr_2 *sex_2 *ida_2 *prof_7

Estamos vivendo uma tribulação coletiva e ao sairmos dela estaremos muito modificados existirá um novo_normal.

**** *ind_0810 *estr_1 *sex_2 *ida_3 *prof_14

Um tanto ansiosa, as vezes triste. Contudo, sou otimista.

**** *ind_0813 *estr_1 *sex_1 *ida_1 *prof_7

A pandemia gerou estresse em quase todos os aspectos sejam eles profissionais ou pessoais.

**** *ind_0817 *estr_2 *sex_2 *ida_3 *prof_12

As vezes muito nervosa quando vejo as estatísticas. Se não soubesse ficaria bem.

**** *ind_0825 *estr_2 *sex_2 *ida_3 *prof_7

Preocupada, triste, estressada.

**** *ind_0826 *estr_2 *sex_2 *ida_3 *prof_7

Impotente.

**** *ind_0829 *estr_2 *sex_2 *ida_2 *prof_14

Exausta psicologicamente com taquicardia e esforço respiratório pós covid.

**** *ind_0831 *estr_2 *sex_2 *ida_1 *prof_7

Sob tensão diária.

**** *ind_0832 *estr_2 *sex_2 *ida_2 *prof_7

Estresse nas situações_cotidianas a necessidade de higienização constante.

Estresse_no_trabalho por conta da paramentação e desparamentação, higienização do equipamento a cada exame_realizado.

**** *ind_0833 *estr_1 *sex_1 *ida_3 *prof_7

Me sinto bem e consciente da pandemia. Colaborando com os meus procedimentos grupo_de_risco.

**** *ind_0834 *estr_2 *sex_2 *ida_1 *prof_11

Tive sintomas de covid, mas testes_negativos, apesar de os médicos acreditarem que foram falsos_negativos. Isso não foi contemplado.

**** *ind_0836 *estr_2 *sex_2 *ida_1 *prof_14

Muito confusa em relação ao futuro.

**** *ind_0837 *estr_2 *sex_2 *ida_1 *prof_14

Me sinto exausta, desestimulada, nunca trabalho tanto na minha vida. No último mês, na minha região a procura relacionado a covid aumentaram muito. Eu não como direito, não sou reconhecida, e não recebo nem insalubridade. Morro de medo levar isso para minha_casa.

**** *ind_0840 *estr_2 *sex_2 *ida_3 *prof_7

Impotente e triste de perceber que não temos luz no final do túnel cada vez mais longo.

**** *ind_0844 *estr_1 *sex_2 *ida_2 *prof_7

Grande estresse por ausência de férias no SUS-SES.

**** *ind_0845 *estr_1 *sex_2 *ida_3 *prof_7

Desolada com o rumo do governo e a falta de cuidados das autoridades para com os profissionais_de_saúde. Mais não é surpresa já sabíamos disso, basta observar o que aconteceu na saúde em janeiro e fevereiro de 2020. Mas somos acomodados e mais uma vez reagimos e nos movimentando de forma inexpressiva, anestesiados.

**** *ind_0846 *estr_1 *sex_2 *ida_3 *prof_11

Insegura com as tomadas de decisão do governo.

**** *ind_0848 *estr_2 *sex_2 *ida_2 *prof_11

Temerosa.

**** *ind_0849 *estr_1 *sex_1 *ida_2 *prof_7

Não tive mudanças em relação ao período antes da pandemia.

**** *ind_0850 *estr_2 *sex_1 *ida_2 *prof_7

Muito deprimido.

**** *ind_0853 *estr_2 *sex_2 *ida_1 *prof_9

Passei a fumar mais na época de quarentena, mas ouvi colegas dizendo que conseguiram parar de fumar por medo da covid. Reações diferentes.

**** *ind_0854 *estr_1 *sex_1 *ida_2 *prof_9

A única questão que não fez sentido neste Questionário foi sobre o epi. Na rede privada, tenho epi adequado. Na rede_pública, não tento epi para trabalho. Preciso levar todo o meu epi para ter alguma segurança no atendimento odontológico.

**** *ind_0855 *estr_2 *sex_2 *ida_2 *prof_5

Com o trabalho adquiri Síndrome de Renaud tenho Transtorno de Ansiedade Generalizada me sinto depressiva e desmotivada, preciso de auxílio de medicamentos tarja preta. Sempre fui alegre e dinâmica, mas comecei a apresentar ansiedade evoluiu para síndrome_do_pânico e hoje não consigo mais trabalho na rede_hospitalar.

**** *ind_0857 *estr_2 *sex_2 *ida_2 *prof_1

Medo e exaustão.

**** *ind_0858 *estr_2 *sex_1 *ida_2 *prof_7

Desamparado pelas ações governamentais contra a ciência.

**** *ind_0859 *estr_1 *sex_2 *ida_1 *prof_14

Nesse momento de pandemia, vejo claramente que nós os profissionais_de_saúde, não estamos preparados para uma pandemia, devemos ser mais valorizados, com salários justos e carga horária de trinta horas semanais, principalmente na rede_privada. Que isso sirva de apoio para nós, que deixamos nossas famílias para cuidado da de vocês, e com muito prazer, mas que merecemos ser valorizados.

**** *ind_0860 *estr_1 *sex_1 *ida_2 *prof_7

Um pouco ansioso.

**** *ind_0861 *estr_2 *sex_2 *ida_2 *prof_14

Acho que deveria receber 1 salário_mínimo, o que não estão pagando.

**** *ind_0863 *estr_1 *sex_2 *ida_2 *prof_6

Senti dificuldade em responder algumas questões por falta de opção para quem não teve covid nem é do grupo_de_risco.

**** *ind_0867 *estr_2 *sex_2 *ida_1 *prof_11

Me sinto abandonada pelo estado. Sem políticas_públicas efetivas para lidar com a pandemia. Um governo_federal totalmente despreparado e criminoso. Está sendo adoecedor.

**** *ind_0869 *estr_1 *sex_2 *ida_3 *prof_7

Na primeira fase muito medo_morrer. Depois me senti_mal de não participar da vida do hospital. Mesmo podendo ficar em_casa por ter 60_anos, optei por trabalho_presencial. Passei para a Direção. Cuidado das internações, vou aos setores, falo com médicos e enfermeiros. Sigo as orientações do ccih quanto ao epi correto para cada lugar que vou. Hoje me sinto melhor, tenho um medo controlado de ter covid, me cuidado, mas não fiquei imobilizada. Penso no hospital o tempo todo, parece que nada existe mais, não consigo mais planejar nada, viagens, encontros. Vida suspensa. O que me irrita muito são as pessoas se comportarem como se a vida tivesse voltado ao normal no rio e em niterói.

**** *ind_0871 *estr_1 *sex_2 *ida_3 *prof_7

Certamente é uma situação muito difícil. Não atendo em setores de pacientes com sintomas_respiratórios, creio que por isso não me sinta estressada no ambiente_de_trabalho, já que a instituição_pública na qual atuo está seguindo todos os protocolos de prevenção à covid.

**** *ind_0873 *estr_1 *sex_2 *ida_2 *prof_11

Diálogo em_casa com outras pessoas que fazem parte de sua convivência diária.

**** *ind_0875 *estr_2 *sex_2 *ida_1 *prof_14

Sobrecarregada.

**** *ind_0876 *estr_1 *sex_2 *ida_2 *prof_7

Cansada, termino o dia exausta.

**** *ind_0880 *estr_1 *sex_2 *ida_2 *prof_5

Precisei ir ao psiquiatra pela primeira vez, por estar entrando em crise_de_pânico, no início da pandemia. Estou em uso_de_medicação e sem nenhum sinal de ansiedade ou pânico, bem controlada para seguir meu compromisso de trabalho.

**** *ind_0882 *estr_2 *sex_2 *ida_2 *prof_9

Sem ver muita luz no fim do túnel.

**** *ind_0883 *estr_1 *sex_2 *ida_1 *prof_3

Eu me sinto sobrecarregada. Tive que acumular, para além da jornada de trabalho_formal, outras atividades do cotidiano como ir ao mercado, à feira, à farmácia. Essas atividades estavam distribuídas com os outros membros do núcleo família, que eu preferi manter dentro de_casa para evitar a exposição ao covid por serem do grupo de risco.

**** *ind_0885 *estr_2 *sex_1 *ida_2 *prof_3

É um momento complicado quenos deixa frágil e vulnerável emocionalmente.

**** *ind_0886 *estr_2 *sex_2 *ida_2 *prof_14

Fiquei neurótica muito medo pegar covid e morrer por ansiedade.

**** *ind_0893 *estr_1 *sex_2 *ida_1 *prof_3

Tentando tirar proveito de maior tempo com meus filhos.

**** *ind_0895 *estr_2 *sex_1 *ida_1 *prof_7

Me sinto perdido, como se essa situação não fosse passar nunca.

**** *ind_0899 *estr_2 *sex_2 *ida_2 *prof_14

Sou da área de saúde a quase 20_anos e nunca me senti tão abandonada, sem informação e vulnerável. Nunca precisei de antidepressivos, minha_vida_profissional nunca havia afetado minha_vida_pessoal. A insegurança_de_trabalho com o desconectado é muito ruim.

**** *ind_0908 *estr_2 *sex_2 *ida_1 *prof_3

Tenho crise_de_ansiedade todos os dias em vários momentos do dia. Acho que vou morrer. Taquicardia, falta_de_ar, dor_no_peito, sentimento de angústia e dor. Enfim tem dias que são mais brandos, tem dias que são insuportáveis. Eu estou de saída do hospital por alguns motivos e aceitei um emprego de home_office, pois sou asmática que tem pressão_baixa, tenho mãe hipertensa asmática, tenho um pai idoso e diabético. E esse outro Emprego me trará mais qualidade_de_vida e segurança. Mesmo assim a todo instante tenho medo e pânico.

**** *ind_0909 *estr_1 *sex_2 *ida_2 *prof_7

O cansaço_físico e mental está maior na pandemia.

**** *ind_0915 *estr_1 *sex_1 *ida_2 *prof_7

Confiante que o pior já passou.

**** *ind_0917 *estr_1 *sex_2 *ida_2 *prof_3

A minha vida não sofreu grandes mudanças, mas sei que sou uma exceção.

**** *ind_0918 *estr_1 *sex_1 *ida_2 *prof_3

Me sinto meio abandonado e sobre carregado, visto que não adoeci, e fiquei muito sobrecarregado no início da Pandemia.

**** *ind_0919 *estr_2 *sex_2 *ida_1 *prof_4

Este momento que vivemos tem me ensinado que a vida é efêmera e que podemos ir ou perder alguém querido com facilidade. Por isso tenho fortalecido minha mente e coração para lidar com essas dores. Tenho lido e me exercitado fisicamente para controlar o estresse que vem com força e muitas vezes inesperadamente. Estar sozinha tem me dado a possibilidade do reencontro comigo mesma.

**** *ind_0921 *estr_1 *sex_2 *ida_1 *prof_3

Estou mais_tranquila agora, depois passei por sintomas do COVID e fiquei bem e com igg_positivo.

**** *ind_0926 *estr_2 *sex_2 *ida_2 *prof_5

Na minha visão não adianta ficar_em_casa pois a saúde_mental da população está se esvaindo ainda mais com notícias distorcidas da televisão aberta que traz pânico nas pessoas.

**** *ind_0928 *estr_1 *sex_1 *ida_1 *prof_5

Me sinto mais confiante e menos preocupado pois já sabemos lidar com a grande maioria das situações. Não tenho mais medo pegar a doença pois já peguei e criei anticorpos.

Me sinto confiante em como tratar os pacientes acometidos por covid.

Ainda estou muito preocupado com as consequências de saúde da população e como a economia irá se comportar.

**** *ind_0929 *estr_2 *sex_2 *ida_2 *prof_5

Ansiedade alta hoje.

**** *ind_0930 *estr_2 *sex_2 *ida_2 *prof_5

Emocionalmente instável.

**** *ind_0931 *estr_2 *sex_1 *ida_1 *prof_5

Mesmo não sendo o meu caso, me revolta ver profissionais_de_saúde trabalho sem plano de carreira, baixa_remuneração e principalmente com sem vínculo_empregatício.

**** *ind_0934 *estr_2 *sex_1 *ida_1 *prof_5

Fisioterapeutas não tem valor nenhum, salários_baixos, poucos epi, sem reconhecimento_social ou de mídia. Infectei a mim e minha família para muitas das vezes estar sem salário.

**** *ind_0937 *estr_1 *sex_2 *ida_1 *prof_5

Estamos em um momento que muitas das vezes não conseguimos proporcionar uma saúde mental ideal para nossos filhos, um dilema envolve o convívio entre pais e avós. Ou seus filhos ficam com eles sem você, ou fica com você e sem eles. Não encontramos quem cuide dos nossos filhos, se a provação é pelos avós. Não querem se arriscar a cuidado de nossos filhos e pegarem a doença e eles filhos não aceitam ficar sem os avós. Estamos sendo massacrados emocionalmente. Muitos não sabem se temem mais a doença física ou a emocional. A pressão da equipe que lida diretamente com os covid está irreal, mesmo aqueles que já estavam acostumados com pacientes críticos. Precisamos repensar e agir rápido com a medicina do trabalho de forma mais eficaz, verdadeira, humanizada, apoiadora. É uma área apagada, morta, insensível e sem destaque, ultrapassada na maior parte do país e essa é a hora de dar a reviravolta e mostra a excelência da área. Oremos por dias melhores e abençoados.

**** *ind_0938 *estr_1 *sex_2 *ida_2 *prof_5

Quem pode ficar_em_casa, fique.

**** *ind_0939 *estr_1 *sex_2 *ida_1 *prof_5

Já estive mais tensa.

**** *ind_0940 *estr_1 *sex_1 *ida_1 *prof_5

Eu me mantive tranquilo, pois compreendo que em caso de descontrole o maior prejudicado seria eu.

**** *ind_0941 *estr_1 *sex_1 *ida_2 *prof_5

Satisfeito por ser e fazer parte da solução para muitas pessoas que ficaram doentes.

**** *ind_0943 *estr_1 *sex_2 *ida_2 *prof_3

Me sinto extremamente triste, sozinha na multidão.

**** *ind_0948 *estr_1 *sex_2 *ida_2 *prof_3

Poderiam investigar sobre a demanda de tarefas dos profissionais no trabalho e as atividades domiciliares, sobrecarga principalmente das mulheres, trabalho no hospital, cuidado da_casa, cuidado dos filhos, suporte a família. Isso tem me causado muito estresse.

**** *ind_0953 *estr_1 *sex_2 *ida_2 *prof_1

Como profissional_da_saúde me sinto desvalorizado, sem apoio institucional e ou governamental. Totalmente desmotivada.

**** *ind_0957 *estr_1 *sex_2 *ida_2 *prof_5

Eu estou com suspeita de covid, fiz o swab mas o resultado ainda não saiu. Me sinto chateada de não poder sair, mas sinto que vou me sentir culpada se ignorar esta recomendação é poder ser um meio de transmissão.

**** *ind_0958 *estr_2 *sex_2 *ida_2 *prof_6

Me sinto muito desgastada por ter que decidir individualmente questões que são coletivas.

**** *ind_0959 *estr_2 *sex_2 *ida_2 *prof_5

Conciliar trabalho_remoto, lactação, cuidados com a rotina da_casa e outros cuidados_pessoais sem ajuda é extenuante e demandam atenção especial pela equipe_de gestão_de_pessoal em período de isolamento_social.

**** *ind_0967 *estr_2 *sex_2 *ida_1 *prof_3

Para quem atua na linha_de_frente no combate ao covid, como profissional_de_saúde, gera bastante estresse, pois a mídia nem sempre passa informações reais, por que digo isso. Durante o pico da pandemia devido_a inúmeras_informações_ cruzadas, tivemos mais atendimentos lotando as emergências, com pacientes com estresse, crise_de_ansiedade, do que com sintomas do covid, quando se trabalho na área_da_saúde, temos que está ciente de que há também um preconceito de família, vizinhos, amigos que não atuam em hospitais. Por medo estarmos infectados. Nesse período de isolamento_social, todo suporte é fundamental para nós que atuamos na linha_de_frente.

**** *ind_0970 *estr_1 *sex_2 *ida_2 *prof_3

Me sinto insegura, com medo do que está por vir daqui para frente.

**** *ind_0971 *estr_1 *sex_2 *ida_1 *prof_5

Me sinto confiante e disposta a desempenhar meu trabalho.

**** *ind_0973 *estr_1 *sex_1 *ida_2 *prof_3

Tranquilo.

**** *ind_0974 *estr_1 *sex_2 *ida_2 *prof_2

Tive momentos de estresse por causa da pandemia e desenvolvi um refluxo por isso. Medito é isso ajuda muito. Os momentos de estresse passaram e agora há um desânimo, cansado do isolamento_social. Trabalho com meio ambiente então sou um biólogo que não é da área_da_saúde.

**** *ind_0977 *estr_2 *sex_2 *ida_2 *prof_7

Sentimento de culpa por parentes contaminados por covid.

**** *ind_0978 *estr_2 *sex_2 *ida_3 *prof_14

Fiquei com pânico mesmo de não conseguir sair ou abrir a porta de minha_casa com isso estou com depressão sendo medicada por psiquiatra e com medo da alta. Agora tenho apoio de minha irmã, mas a ansiedade me faz ter falta_de_ar ficar sufocada, gelada principalmente o medo se ficar desempregada com essa idade. Meus pensamentos me levam a chorar muito.

**** *ind_0980 *estr_1 *sex_2 *ida_1 *prof_15

Sinto muita falta de poder compartilhar momentos com família. Em especial meu afilhado que nasceu durante esse período e tive contato por vídeo.

**** *ind_0985 *estr_1 *sex_1 *ida_2 *prof_7

Estou bem apesar das restrições na vida_social e afetiva que geram alguma ansiedade. Me conformei que precisamos aguardar e seguir trabalho.

**** *ind_0987 *estr_2 *sex_2 *ida_1 *prof_14

Inicialmente achei quefosse morrer, tenho doença de crohn e faço uso_de_imunobiológicos. Hoje já trabalho a minha cabeça de que o contato com o vírus será inevitável, então estou mãos confiante quanto a isso, embora temerosa, mas bem menos que antes.

**** *ind_0988 *estr_1 *sex_2 *ida_2 *prof_3

Me sinto triste por não poder circular na_rua, ir ao shopping, encontrar amigos. Não vejo a hora dessa pandemia acabar e poder encontrar todo mundo de novo e abraçar.

**** *ind_0989 *estr_2 *sex_2 *ida_1 *prof_3

Peguei covid no início, em abril. As duas instituições que trabalho não me testaram.

**** *ind_0990 *estr_2 *sex_2 *ida_1 *prof_3

Me sinto esgotada mentalmente, commedo, apesar de morar sozinha agora, tenho uma mãe doente, e se ela precisar de mim, com certeza irei, meu setor é de paciente positivos e suspeita de covid , o medo é grande.

**** *ind_0992 *estr_1 *sex_2 *ida_2 *prof_7

Aproveitado para ler e estudar e, na medida do possível, não pensar em coisas ruins.

**** *ind_0994 *estr_1 *sex_1 *ida_3 *prof_7

Tenho enfrentado de maneira adequada como em outros momentos de minha_vida adulta.

**** *ind_0995 *estr_2 *sex_2 *ida_1 *prof_5

Momento muito delicado, com muitas preocupações, pensamentos na cabeça, que passa quando faço algo relaxante exercícios_físicos e meditação ou com a psicóloga. Ansiedade maior do que costumava ficar, insônia e tomando mais medicamentos relaxantes do que antes da pandemia.

**** *ind_0996 *estr_1 *sex_2 *ida_3 *prof_7

Mais_tranquila.

**** *ind_0998 *estr_1 *sex_2 *ida_1 *prof_14

Adorei participar dessa pesquisa, trabalho diretamente cuidado dos pacientes acometidos pelo covid e conseguir analisar com mais delicadeza e amor coisas cotidianas que as vezes nem ligamos muito e que ao nos vermos enfrentando um inimigo invisível damos mais valor a família, o estar no convívio precioso de nossos entes queridos. A pior parte para mim nessa pandemia era o paciente ficar sozinho em um quarto onde entrava todos os profissionais paramentados e que as vezes via nossos olhos, e a melhor parte era ver as chamadas de vídeo emocionantes de cada família que via que seu parente estava bem e não mais ouvia de um médico que estava tudo bem.

**** *ind_0999 *estr_1 *sex_1 *ida_3 *prof_15

Vejo com preocupação este momento, porém não deixo de fazer nada que preciso fazer tipo, trabalho com terceiros e conviver com dependente químico que é a essência do meu trabalho e tomo todas as medidas de prevenção possíveis. Obrigado pela oportunidade.

**** *ind_1003 *estr_1 *sex_2 *ida_3 *prof_14

Insegurança e medo. Profissionais_de_saúde sejam mais valorizados e tratados com mais respeito.

**** *ind_1007 *estr_2 *sex_2 *ida_1 *prof_3

Muito medo da morte também de pessoas próximas, muita angustia e irritação. Sentimento de prisão enquanto a vida volta a um novo_normal lá fora.

**** *ind_1008 *estr_1 *sex_1 *ida_2 *prof_14

Todos.

**** *ind_1012 *estr_1 *sex_2 *ida_2 *prof_14

Ansiosa.

**** *ind_1015 *estr_1 *sex_2 *ida_1 *prof_3

Estresse, muito_cansaço, acordar durante a noite, dormir mal.

**** *ind_1016 *estr_1 *sex_1 *ida_2 *prof_14

Me sinto bem,e não tenho nada a sugerir.

**** *ind_1017 *estr_2 *sex_2 *ida_2 *prof_3

Com medo contrair e chegar até a forma mais grave e morrer ou pior, passado para meus filhos, mãe ou esposo ou até mesmo conhecidos e eles agravarem e morrerem.

**** *ind_1019 *estr_2 *sex_2 *ida_2 *prof_3

Muito angustiada , sem perspectivas visto como a saúde está sendo tratada com descaso.

**** *ind_1020 *estr_2 *sex_2 *ida_2 *prof_14

Não estou satisfeita no cti, como era na estratégia_da_família.

**** *ind_1021 *estr_1 *sex_2 *ida_2 *prof_3

Gostaria de trabalho mais próximo da minha residência. Trabalho no bombeiro e trabalho há mais de 150_quilometros de_casa. Isso me causa muito estresse e ansiedade.

**** *ind_1022 *estr_2 *sex_2 *ida_2 *prof_14

Sozinha.

**** *ind_1023 *estr_2 *sex_2 *ida_3 *prof_15

Muito inquieta sem controle de meus dias. A angústia e grande o medo e frequente dia de sim e dia de não. Tento ser calma mas não é fácil se correr o bicho_pega e se ficar o bicho_come.

**** *ind_1024 *estr_1 *sex_2 *ida_3 *prof_15

Sem perspectiva futura.

**** *ind_1026 *estr_2 *sex_2 *ida_2 *prof_15

Ver o povo abandonado, sem orientação pelas_ruas. Desorientação das pessoas geradas em grande parte pela forma como as notícias são passadas. Jornalistas reduzindo vidas a números apenas. Ano_eleitoral, apenas isso importa.

**** *ind_1027 *estr_1 *sex_1 *ida_3 *prof_7

Confiante e trabalho visando um futuro_melhor.

**** *ind_1028 *estr_2 *sex_2 *ida_1 *prof_3

Exausta, muito trabalho, inclusive como docente.

**** *ind_1029 *estr_1 *sex_2 *ida_3 *prof_7

Há pacientes com testes negativos, no meu caso, porém com imagens em tórax e quadro clínico muito sugestivo. O questionário não contempla essa opção. Gostei muito da ideia e do questionário. Sinto que a situação da pandemia no país está fora de controle há muitos meses e a população sem esclarecimentos suficientes por parte das três esferas governamentais. Há muita desinformação. A imprensa oficial, especialistas e pesquisadores têm suprido em parte essa lacuna. Parabéns a todos os colegas. Estou divulgando o questionário.

**** *ind_1036 *estr_1 *sex_2 *ida_2 *prof_11

O medo e a incerteza ainda perturba, mas vejo a esperança como força maior para suportar.

**** *ind_1037 *estr_2 *sex_2 *ida_2 *prof_7

Com medo pela falta de respeito as regras pelas pessoas.

**** *ind_1045 *estr_1 *sex_2 *ida_3 *prof_7

Me sinto em compasso de espera, vivendo mais o presente e fazendo poucos planos_futuros pois enquanto não houver vacina a pandemia continua.

**** *ind_1046 *estr_1 *sex_2 *ida_2 *prof_7

O medo está maior que a doença, temos medo morrer em acidente de trânsito, mas dirigimos.

**** *ind_1047 *estr_1 *sex_2 *ida_3 *prof_7

Insegura por trabalho presencialmente neste momento. Principalmente porque fui obrigada a retornar. Medo principalmente do ambiente hospitalar e deslocamentos, mora em outra cidade. Irritada com políticas sociais negligenciadas, e o estado de negação de algumas pessoas e dirigentes.

**** *ind_1048 *estr_1 *sex_1 *ida_1 *prof_14

Sinto uma falta de respeito com nós profissional. A onde se cuidamos e tem muitas pessoas que não se cuida, pagamos condução para trabalho e ainda tem pessoas sem_máscara e não se prevenir.

**** *ind_1049 *estr_2 *sex_2 *ida_1 *prof_9

Tomei algumas medidas como acordar mais cedo e programar meu dia com antecedência. Procurei por amigos. Procurei um trabalho novo que me gerou mais motivação. Iniciei uma medicação para controle de ansiedade. Iniciei meditação Intensifiquei leitura para autoconhecimento Infelizmente parei a psicóloga por questões_financeiras.

**** *ind_1050 *estr_2 *sex_2 *ida_2 *prof_5

Me sinto cansada.

**** *ind_1052 *estr_2 *sex_2 *ida_3 *prof_7

Desanimada.

**** *ind_1054 *estr_2 *sex_2 *ida_2 *prof_3

Gostaria de marcar a importância dos profissionais de gestão e da vigilância, que sofrem grande pressão para produzir resultados mas durante a pandemia me parecem invisíveis. Reconheço o brilho e importância dos que estão na assistência, mas na gestão e na vigilância, a pressão não é menor nosso dia a dia nos exige agilidade na produção de informações com qualidade para subsidiar a tomada de decisões e apoiar os que estão na linda_de_frente. A pandemia se conhece através desse trabalho invisível, pouco valorizado e noticiado. Também temos medo adoecer, levar o vírus para casa, perder pessoas da equipe que adoecem e precisamos permanecer firmes. Não ter visibilidade nos faz parecer sem relevância e desvalorizados.

**** *ind_1060 *estr_2 *sex_2 *ida_2 *prof_14

Insegura.

**** *ind_1061 *estr_2 *sex_2 *ida_1 *prof_3

Com mais crise_de_ansiedade, angustiada e com medo pelos que amo e não posso ver.

**** *ind_1062 *estr_1 *sex_2 *ida_2 *prof_11

Sinto saudades do contato com as pessoas, de abraçar e beijar meias amigos e família, de mergulhar_no_mar.

**** *ind_1063 *estr_1 *sex_2 *ida_2 *prof_3

Maior suporte_psicológico para os profissionais_da_saúde.

**** *ind_1065 *estr_1 *sex_1 *ida_3 *prof_7

Me aflige o pequeno número_de profissionais_da_saúde de psiquiatria como eu e a maioria descredenciada. Uma sobrecarga grande no momento.

**** *ind_1068 *estr_1 *sex_2 *ida_2 *prof_7

Me sinto cansada de ficar isolada, sinto falta do contato pessoal com família e amigos e de praticar atividade_física coletiva.

**** *ind_1069 *estr_1 *sex_2 *ida_2 *prof_7

Carga de trabalho diminuída, maior tempo_de_sono e muito aproveitamento domiciliar com marido e filhos, além de cuidado da_casa. Dei continuidade aos exercícios_físicos como corrida na praia.

**** *ind_1073 *estr_2 *sex_2 *ida_1 *prof_7

Estou muito sobrecarregada por coordenar disciplinas e exames e cuidado de tarefas domésticas, alimentação e uma criança de dois anos. Ela acha que estou em_casa apenas no papel_de_mãe, não entende que eu trabalho absolutamente compreensível porque antes eu evitava ao máximo trabalho em_casa. Eu e o marido não temos rede de apoio. Tinha compromissos profissionais importantes que não têm prazo para ocorrer mais. Simplesmente não consigo ter nenhuma expectativa nem esperança para o futuro e penso seriamente em ir embora do país apesar de já ter vivido fora e não recomendar isso antes. Esqueço o aniversário dos amigos. Não tenho mais vontade de cuidado de mim.

**** *ind_1078 *estr_2 *sex_2 *ida_2 *prof_7

A ansiedade e preocupação com o futuro são os maiores sentimentos neste momento.

**** *ind_1081 *estr_2 *sex_2 *ida_2 *prof_3

Uma semana antes do início do distanciamento social estava reduzindo a quantidade de medicações para depressão. Com isto tive que voltar a tomar os remédios e fiquei uns meses de licença. Adquiri o covid, fiquei isolada e meu esposo que também adquiriu a doença faleceu em uma semana e eu nem pude participar. Me despedir, pois estava em isolamento. Muito triste.

**** *ind_1084 *estr_1 *sex_2 *ida_2 *prof_7

Sensação de impotência.

**** *ind_1086 *estr_1 *sex_2 *ida_3 *prof_15

A visão de ter o controle sobre a vida é utópico. Podemos aprender a conhecer a nós mesmos e por aí administrarmos a vida ao redor. Senti dificuldade com a última parte do questionário por considerar que eu não tenho nenhum controle sobre a vida. Apenas posso ter controle sobre a minha vontade de querer me conhecer melhor frente as dificuldades que surgem e aprender com elas. A pandemia tem sido um tempo para redirecionar as prioridades, olhar para o outro como se fosse a mim mesmo e agir no grande o planeta e os seres, no pequeno individualidade. Um tempo para repensar a vida, os hábitos. Me sinto segura porque tenho uma alimentação_adequada, assisto ao nascer do sol quase todo dia, durmo cedo, faço exercícios diariamente. Uso a vitamina e também uso a tecnologia ou ciência do plasma.

**** *ind_1088 *estr_1 *sex_2 *ida_1 *prof_3

Me sinto com um medo constante e temendo sempre pelos meus família principalmente minha bebê tenho medo ir aveia ao trabalho mas temos que reaprender a viver nestas circunstâncias

**** *ind_1089 *estr_2 *sex_2 *ida_1 *prof_3

Me sinto muito angustiada e ansiosa.

**** *ind_1091 *estr_1 *sex_1 *ida_3 *prof_7

Época da incerteza.

**** *ind_1098 *estr_2 *sex_2 *ida_2 *prof_11

Me sinto cansada, como se estivesse em alerta constante, o que junto com as demandas do trabalho e necessidade de cuidados com limpeza, alimentação e outros comprometeu muito a qualidade do meu sono, sendo necessário o uso_de_medicamentos prescrito por psiquiatra. Como tenho dor_crônica, a rotina estendida no computador e a falta de limite no tempo de trabalho aumentou muito às crises de dor e fadiga. Mas estou com menos respostas positivas medicações que já utilizava para dor, fazendo com que eu conviva com mais dores.

**** *ind_1100 *estr_2 *sex_2 *ida_2 *prof_14

Me senti irritada e acredito estar relacionado a dificuldade em dormir.

**** *ind_1101 *estr_1 *sex_2 *ida_2 *prof_7

Estou melhor no momento, porém no início da pandemia fiquei muito triste e nervosa.

**** *ind_1102 *estr_1 *sex_1 *ida_2 *prof_3

Inseguro.

**** *ind_1104 *estr_1 *sex_2 *ida_2 *prof_3

Estou bem dentro do possível.

**** *ind_1106 *estr_2 *sex_2 *ida_2 *prof_2

Impotente. Difícil demais estar afastada de meu irmão, mãe, filho, amigos e família, por amar, por respeitar, por não querer gerar riscos e contaminar e constatar a incompetência e irresponsabilidade dos gestores, de todos as esferas_governamentais, verificar que a população, do mundo inteiro, ignora, desrespeita e segue firme contribuindo para disseminação e aumento no número_de_casos de mortes. Impotente. Triste.

**** *ind_1107 *estr_1 *sex_2 *ida_2 *prof_10

Caso eu tenha convívio com idosos e crianças e tenha o covid, como vou me sentir.

**** *ind_1110 *estr_1 *sex_2 *ida_3 *prof_2

Receio de contrair a covid, pela minha idade e principalmente passar para meus filhos e esposo renal_crônico. Mesmo com todo o controle e calma.

**** *ind_1114 *estr_1 *sex_2 *ida_1 *prof_3

Eu estou desnorteada pois testei positivo em abril, e testei positivo novamente em agosto. Com sintomas diferentes.

**** *ind_1115 *estr_1 *sex_2 *ida_2 *prof_2

Suspeito que tive covid em março antes do isolamento, assim como meu filho, mas na época não tinham testes. Nosso Diagnóstico foi traqueite viral. 3 meses depois demos negativo no teste_rápido. Me sinto sob controle mas com terapia.

**** *ind_1117 *estr_2 *sex_2 *ida_3 *prof_3

Ansiosa.

**** *ind_1119 *estr_2 *sex_2 *ida_1 *prof_7

Nesse momento me sinto mais equilibrada após eu e toda minha família já ternos tido o covid e estarmos com sorologia, mesmo sem saber quanto tempo estaremos imunes. Vivi momentos de muitos estresse em abril, maio e junho. Agora estou mais_tranquila, com a diminuição_do número_de_casos no Rio e por já ter tido covid.

**** *ind_1123 *estr_1 *sex_2 *ida_3 *prof_7

Não me senti estressada porém muito triste com toda situação mundial e especialmente no nosso país e especialmente em relação aos menos favorecidos.

**** *ind_1124 *estr_1 *sex_2 *ida_2 *prof_7

Sobrecarregada por afazeres que antes da pandemia não fazia; cansada de conviver com as mesmas pessoas; chateada por não poder encontrar fisicamente e dançar com meus amigos, insegura com relação ao tempo que ainda ficaremos em isolamento; irritada por ter as filhas de volta em_casa já não estavam mais conosco pois elas estão estressadas.

**** *ind_1127 *estr_1 *sex_1 *ida_3 *prof_7

Me sinto bem, tomando as precauções necessárias.

**** *ind_1128 *estr_1 *sex_2 *ida_2 *prof_8

Perguntar sobre se considerou viver ou morrer durante a pandemia. As consequências família, pois o terror psicológico foi intenso.

**** *ind_1130 *estr_1 *sex_2 *ida_2 *prof_7

Me sinto_mal , porque estamos lidando com o desconhecido, e também com informações desencontradas e com inércia e incapacidade de_gestão dos governates.

**** *ind_1131 *estr_2 *sex_2 *ida_2 *prof_9

Me sinto insegura. Não sabemos direito ainda o dano que esta pandemia causou as nossas vidas. depois da vacina é que poderemos retornar a nossa vida de antes e aí sim avaliarmos.

**** *ind_1133 *estr_2 *sex_2 *ida_2 *prof_7

Sinto bem estressada e ansiosa sem saber o tempo que esta fase ainda vai durar. Muitas informações contraditórias e muita polarização também.

**** *ind_1137 *estr_2 *sex_2 *ida_1 *prof_2

Ansiosa.

**** *ind_1139 *estr_1 *sex_1 *ida_2 *prof_7

Me preocupo com a forma que os governantes e empresários_da_saúde lidam com os problemas da pandemia, de forma corrupta e de opressão aos profissionais_de_saúde afetando a todos os pacientes.

**** *ind_1144 *estr_1 *sex_1 *ida_3 *prof_7

Medo de adoecer.

**** *ind_1148 *estr_2 *sex_1 *ida_2 *prof_7

Muito cansado, sobrecarregado, exposto, com medo, subvalorizado

**** *ind_1151 *estr_1 *sex_2 *ida_2 *prof_11

Sobrecarregada profissionalmente. Os atendimentos virtuais ocorrem em horários diversos e fora do planejamento, dado a necessidade premente de vários usuários. Por tratar na maioria dos casos de quadro depressivo e de ansiedade, não consigo deixar de atender.

**** *ind_1152 *estr_2 *sex_2 *ida_1 *prof_7

Cansada.

**** *ind_1153 *estr_1 *sex_2 *ida_2 *prof_7

Triste pela vida não ser como antes.

**** *ind_1154 *estr_1 *sex_2 *ida_2 *prof_7

Insegura.

**** *ind_1155 *estr_1 *sex_2 *ida_2 *prof_7

Tranquila. Tenho fé_em_deus que tudo vai passar da melhor_maneira.

**** *ind_1156 *estr_2 *sex_2 *ida_1 *prof_7

Acredito que os profissionais_de_saúde precisavam de um suporte_psicológico maior, dentro da unidade_de_trabalho. Não é fácil expor-se a risco, ter salários_atrasados, falta_de_recursos, ver colegas e família morrendo.

**** *ind_1157 *estr_2 *sex_2 *ida_1 *prof_14

Me sinto sem rumo, como se estivesse perdida.

**** *ind_1160 *estr_1 *sex_2 *ida_3 *prof_7

Sinto que a situação está fora de controle.

**** *ind_1162 *estr_1 *sex_1 *ida_2 *prof_7

Acho que na minha cidade o pior já passou e por ter sido muito bem conduzida pelo poder_público não acho que teremos segunda_onda.

**** *ind_1163 *estr_1 *sex_2 *ida_3 *prof_7

Ansiosa para o início da vacina.

**** *ind_1164 *estr_2 *sex_2 *ida_3 *prof_7

Insegura.

**** *ind_1166 *estr_1 *sex_2 *ida_2 *prof_7

A sensação nesse momento é de angústia diante da politização do tratamento por muitos médicos, e da roubalheira de muitos prefeitos e governadores. Muito triste tudo isso. Outra coisa que me entristeceu demais são as crianças longe da escola, um prejuízo inestimável para elas, infelizmente.

**** *ind_1167 *estr_1 *sex_2 *ida_2 *prof_7

Tenho aprendido a exercitar a resiliência.

**** *ind_1169 *estr_1 *sex_1 *ida_2 *prof_7

Me sinto bem. Procuro encarar de forma tranquila e positiva.

**** *ind_1172 *estr_1 *sex_2 *ida_2 *prof_7

Tranquila. Tenho fé_em_deus que tudo vai passar da melhor_maneira.

**** *ind_1174 *estr_1 *sex_1 *ida_2 *prof_7

Me sinto bem. Agora é esperar pela vacina.

**** *ind_1179 *estr_1 *sex_2 *ida_2 *prof_14

Temerosa mas com muita fé.

**** *ind_1182 *estr_1 *sex_2 *ida_2 *prof_14

As vezes sinto medo perder pessoas da família e pacientes por covid

É ansiedade por não ter certeza do tratamento_adequado.

**** *ind_1183 *estr_1 *sex_2 *ida_3 *prof_7

Difícil lidar com perdas de colegas da área de saúde ou não, pelo covid.

**** *ind_1185 *estr_2 *sex_2 *ida_3 *prof_7

Muito_bom. Nunca estive melhor. me preocupo com as pessoas, o país e o mundo, como sempre.

**** *ind_1186 *estr_1 *sex_1 *ida_3 *prof_7

Para passar pela pandemia penso que temos que olhar para frente e se desvincular das amarras do passado.

**** *ind_1189 *estr_1 *sex_2 *ida_2 *prof_11

Receoso.

**** *ind_1194 *estr_1 *sex_2 *ida_1 *prof_7

Incerteza.

**** *ind_1196 *estr_1 *sex_2 *ida_2 *prof_11

Cansada.

**** *ind_1197 *estr_1 *sex_1 *ida_1 *prof_7

Incerteza.

**** *ind_1200 *estr_2 *sex_2 *ida_2 *prof_3

Me sinto ansiosa, cansada e preocupada. Apresentei reflexos de estresse na pele. Tendo que fazer tratamento com corticoide tópico sem êxito. Já se passaram 4_meses de tratamento e ainda não melhorei.

**** *ind_1202 *estr_1 *sex_2 *ida_2 *prof_3

Sobrecarga_ de_trabalho por afastamento de colegas. Sentimentos de impotência diante das mortes de muitos colegas_de_trabalho. Medo de ser a próxima_vítima. Medo contaminar minha família. Ausência de reconhecimento pela sobrecarga_de_ trabalho. Ausência de uma gratificação por estar na linha_de_frente. Suspensão_de_férias e folgas neste momento_difícil de estresse e perda dos seus colegas_de_trabalho. Suspensão de direitos_trabalhistas como contagem de triênios, licença_prêmio, perda de adicional_noturno para os profissionais com afastamento por doenças_crônicas ou covid. A falta de informação para a equipe_dos_profissionais afastados_por covid. A não informação sobre os resultados de covid das pacientes na admissão em trabalho de parto e cesáreas. Após suspeitas eram transferidos para cti ou isolamento_social e a chefia sempre omitindo o resultado do covid, alegando ser negativo e vários profissionais sendo afastados_por covid. Apos manipulação dos referidos pacientes. Profissionais contaminados e afastado, sem nenhum suporte da chefia e direção em oferecer ajuda ou, pelo menos, ligar para saber do quadro. A única preocupação era a diminuição dos profissionais. Em nenhum momento foi suspenso cirurgias_eletivas, mesmo com deficit de profissionais. Proibição pela diretora médica e da enfermagem do uso_de_máscaras em pacientes não suspeitas para evitar pânico nas pacientes. Controle numérico de máscaras cirúrgicas, luvas, álcool a 70 por cento e tocas sob alegações que os profissionais desviariam roubando por histeria.

**** *ind_1203 *estr_1 *sex_2 *ida_2 *prof_7

Não consegui marcar fadiga. A pandemia traz uma sensação que a vida não é real. Parece um filme que não acaba nunca.

**** *ind_1204 *estr_1 *sex_2 *ida_1 *prof_14

Me sinto bem mas com receio de manter a minha rotina.

**** *ind_1208 *estr_1 *sex_2 *ida_2 *prof_11

Estou tentando dar conta do trabalho da família e de todo estresse.

**** *ind_1211 *estr_1 *sex_2 *ida_3 *prof_7

Triste por não poder Encontrar os família e amigos. Também em por não poder fazer viagem. Muito triste pelo sofrimento das pessoas perdendo entes queridos e seus empregos e negócios. Também triste pelo risco de alguma família ou amigo contrair a covid.

**** *ind_1213 *estr_2 *sex_2 *ida_2 *prof_7

Cansada e sobrecarregada.

**** *ind_1215 *estr_1 *sex_2 *ida_1 *prof_14

Me senti bem graças a deus, apesar de me preocupar com minha família, principalmente mãe e filho, mais além do trabalho me envolvi muito com trabalho_voluntário, como montagem e entrega de cestas_básicas, fazendo as campanhas de arrecadação de alimentos por rede_social.

**** *ind_1216 *estr_1 *sex_2 *ida_3 *prof_7

Faço apenas consultório. Me sinto exausta no final do dia, cansada, estressada, higienização das salas a cada paciente, e medo chegar em_casa e levar o vírus para casa.

**** *ind_1222 *estr_1 *sex_2 *ida_2 *prof_7

Muito sobrecarregada e pressionada.

**** *ind_1225 *estr_1 *sex_2 *ida_2 *prof_7

Sinto cansada.

**** *ind_1229 *estr_1 *sex_1 *ida_2 *prof_7

Tranquilo.

**** *ind_1234 *estr_1 *sex_2 *ida_3 *prof_7

Me sentindo assustada por saber tão pouco sobre essa pandemia não encontrando suporte científico mais específico e confiável pois o que mais me assusta é o jogo de interesses pessoais e dos governantes no que tange a saúde mundial.

**** *ind_1235 *estr_1 *sex_2 *ida_2 *prof_7

Esperançosa.

**** *ind_1238 *estr_2 *sex_2 *ida_3 *prof_7

Insegura e irritada.

**** *ind_1241 *estr_2 *sex_2 *ida_2 *prof_7

Neste momento, me sinto cansada.

**** *ind_1242 *estr_2 *sex_2 *ida_2 *prof_7

Impotente e com raiva dos governantes.

**** *ind_1243 *estr_2 *sex_2 *ida_2 *prof_7

Por vezes apavorada. Triste por todos. Voltei_me para meu interior. Projetos pessoais, meditação e deus.

**** *ind_1246 *estr_1 *sex_1 *ida_3 *prof_7

Sinto_me normalmente diminuir em 50 por cento meu atendimento em meu consultório devido_a queda de pacientes.

**** *ind_1248 *estr_1 *sex_2 *ida_2 *prof_7

Gostaria de ter um governante mais humano e responsável.

**** *ind_1250 *estr_2 *sex_2 *ida_2 *prof_7

Medo.

**** *ind_1251 *estr_1 *sex_2 *ida_2 *prof_11

Muito cansada.

**** *ind_1252 *estr_2 *sex_2 *ida_2 *prof_7

Me sinto cansada e estressada porque trabalho durante todo o tempo. Não consigo enxergar o fim da pandemia e reinício de um novo normal.

**** *ind_1253 *estr_1 *sex_2 *ida_1 *prof_14

Sinto que, por mas cuidados que temos, ainda assim estamos nos contaminado e como se todo aquele esforço para se proteger não adiantasse de nada.

**** *ind_1256 *estr_2 *sex_2 *ida_2 *prof_2

Sinto com mais_tranquilidade mas preocupada com o aumento de casos no rio_de_janeiro.

**** *ind_1257 *estr_1 *sex_1 *ida_3 *prof_7

Sobre cumprir compromissos_financeiros, dívidas.

**** *ind_1260 *estr_2 *sex_2 *ida_2 *prof_2

queria a minha_vida de_volta.

**** *ind_1261 *estr_2 *sex_2 *ida_2 *prof_7

Cansada.

**** *ind_1262 *estr_2 *sex_2 *ida_3 *prof_7

Triste, por ser idosa e não poder contribuir com esse momento desesperador em que estamos passando, sinto falta do meu trabalho, além de não poder visitar e abraçar filhos e netos e viver socialmente junto aos outros família e amigos. Poderíamos ao menos fazer telemedicina instrucional para ajudar e ocupar a cabeça.

**** *ind_1266 *estr_1 *sex_1 *ida_3 *prof_7

Solidão.

**** *ind_1267 *estr_1 *sex_2 *ida_3 *prof_7

Apreensiva.

**** *ind_1269 *estr_2 *sex_2 *ida_3 *prof_7

Insegura.

**** *ind_1271 *estr_1 *sex_1 *ida_2 *prof_2

Tem sido importante o contato com a profissional_de_assistência_social de minha unidade, mas o contato com o setor de recursos_humanos é menos frequente. Espero que o uso do sistema_eletrônico de informação. Tive outros problemas_de_saúde com provável ligação à covid, mas o plano_de_saúde e minha instituição_de_trabalho oferecem o suporte necessário.

**** *ind_1274 *estr_1 *sex_2 *ida_2 *prof_4

Insegura por ter que sair de_casa, usar, transporte_público, bem difícil mais é necessário.

**** *ind_1279 *estr_1 *sex_1 *ida_3 *prof_7

Consciente da situação.

**** *ind_1283 *estr_1 *sex_2 *ida_3 *prof_7

Estou afastada do meu plantão de neonatologia pelo decreto e faço ambulatório de transmissão_vertical com proteção e me sinto muito_tranquila em relação a isso.

**** *ind_1284 *estr_1 *sex_1 *ida_2 *prof_4

Um pouco estressado e temeroso diante da possibilidade do uso de transporte_público. Gostaria de saber se haverá teste para os participantes.

**** *ind_1285 *estr_2 *sex_2 *ida_2 *prof_7

A falta de respeito ao profissional_de_saúde. Fui afastada por suspeita de covid pelo hospital público de buzios e o mesmo descontou da folha_de_pagamento este plantão que fui afastada.

**** *ind_1286 *estr_2 *sex_2 *ida_2 *prof_2

Consigo ver a importância do isolamento_social e a gravidade do problema, mas tendo como objetivo a preservação da saúde de parentes e amigos, além da minha saúde, convivo sem histeria e pessimismo. Deveriam ter perguntas sobre mudança de algum comportamento com vizinhos, colegas_de_trabalho, porteiros. E da relação com a chefia imediata com relação ao trabalho remoto e com o manejo de sintomas e encaminhamento à saúde_do_trabalhador dos profissionais.

**** *ind_1288 *estr_2 *sex_2 *ida_3 *prof_7

E sinto insegura com o.imenso e o futuro.

**** *ind_1293 *estr_2 *sex_2 *ida_1 *prof_7

Perdi meu pai com menos de 1 mês para complicações do covid.

**** *ind_1295 *estr_2 *sex_2 *ida_3 *prof_7

Me sinto sob_pressão. Estou procurando fazer o melhor, mas está difícil. Fica mais fácil quando sou sustentada pelos meus princípios religiosos sou Espírita, porém ainda deixo a desejar em relação a manter a sintonia. Preciso trabalho, cuidado do meu neto de 4_anos minha filha é médica, trabalho todos os dias, e a creche está fechada, da minha_mãe de 93_anos, cada um em sua casa, e do meu marido de 77_anos, na nossa_casa, além dos cuidados com o lar auxiliares tiveram covid.

**** *ind_1296 *estr_1 *sex_2 *ida_2 *prof_11

As vezes triste por estar longe dos amigos e ter que desenvolver sozinha atividades que seriam realizadas em grupo, com alegria. Muito reflexiva sobre prioridades e rumo que darei a minha vida.

**** *ind_1299 *estr_1 *sex_2 *ida_2 *prof_7

Resiliente.

**** *ind_1301 *estr_1 *sex_2 *ida_2 *prof_7

Eu me sinto confusa com tantas informações, mas tenho tentado fazer a minha parte e orientar os mais próximos.

**** *ind_1306 *estr_1 *sex_1 *ida_3 *prof_7

Devido_a baixa dos atendimentos pediátricos, fui dispensado. Estou desempregado desde maio.

**** *ind_1309 *estr_2 *sex_2 *ida_3 *prof_7

Insegura.

**** *ind_1312 *estr_1 *sex_2 *ida_2 *prof_8

No meu entender a questão das informações e treinamento sobre o covid onde trabalho, foi o ponto crucial que levou a contaminação de colegas. E a questão do fornecimento tardio dos epi. O fato de ser também bióloga me ajudou muito a não me contaminar e a ter mais cuidados. A falta de informações dos profissionais de saúde foi crucial p contaminação de muitos.

**** *ind_1313 *estr_2 *sex_2 *ida_1 *prof_6

Me sinto cansada e com flutuação no humor.

**** *ind_1315 *estr_2 *sex_2 *ida_2 *prof_8

Com receio de ser contaminada, contrair a covid, contaminar meus filhos, ter complicações e necessitar de internação em cti. Esse ciclo me apavora mas, tem que trabalho diariamente, sem escalas, sem folgas e com muitas cobranças.

**** *ind_1316 *estr_1 *sex_1 *ida_1 *prof_10

Inseguro com o que virá para frente.

**** *ind_1317 *estr_1 *sex_2 *ida_2 *prof_14

Me sinto ainda insegura.

**** *ind_1318 *estr_1 *sex_2 *ida_3 *prof_16

Tenho sentido falta de sol.

**** *ind_1319 *estr_1 *sex_2 *ida_3 *prof_7

Se usar hidroxicloroquina, sulfato de zinco e azitromicina logo nas primeiras 48 horas de sintomas o vírus não progride no organismo e debelamos os sintomas.

**** *ind_1321 *estr_1 *sex_2 *ida_3 *prof_7

Resolvi voltar a viver na nova normalidade com a pandemia, tomando os cuidados necessários à prevenção e contaminação pelo covid.

**** *ind_1323 *estr_2 *sex_2 *ida_2 *prof_14

Me sinto extremamente estressada devido_a dificuldades em locomoção_para trabalho e não vejo instituição que trabalho se esforçando para ajuda com algumas exceções.

**** *ind_1329 *estr_2 *sex_1 *ida_2 *prof_11

Tirei uns dias de descanso e me encontre mais disposto.

**** *ind_1330 *estr_1 *sex_2 *ida_2 *prof_3

Momento muito difícil que estamos vivendo, muitas perdas, mas também muita confiança em deus.

**** *ind_1332 *estr_1 *sex_2 *ida_2 *prof_3

Me sinto bem, porque sou bem preparada para lidar com toda essa situação, recebo treinamento e palestra. Tenho todos equipamentos necessário para trabalho, sigo corretamente as ordens para não prejudicar quem está a minha volta.

**** *ind_1334 *estr_2 *sex_2 *ida_2 *prof_14

Me sinto acuada diante da falta de educação das pessoas que não seguem as regras de convivência social. E pelo fato do governo e prefeitura se omitirem em diversos fatores em relação a pandemia, como por exemplo, medidas eficazes em relação ao cumprimento das leis.

**** *ind_1336 *estr_2 *sex_2 *ida_2 *prof_3

Os sentimentos são muito diversos. Ao mesmo tempo que sinto que tenho sido resiliente, às vezes bate uma tristeza por tudo que estamos vivendo e passando e o choro vem. Eu mudei muito depois que tive covid. Tenho prestado mais atenção em mim, buscado mais meu autoconhecimento e cuidado da minha mente e corpo. Tenho prestado mais atenção na minha família também. Desacelerei muito e isso foi muito_bom. Ainda tenho receio do contágio, dos meus família também. Mas me sinto muito melhor do que no início de tudo.

**** *ind_1337 *estr_2 *sex_2 *ida_2 *prof_5

Aumento importante da ansiedade, agitação, sintomas gástricos necessitando tratamento medicamentoso e cefaleia frequente.

**** *ind_1338 *estr_2 *sex_1 *ida_1 *prof_3

Abandonado pelas instâncias públicas_de_saúde. Atraso de salário e descumprimento de direitos básicos gera em nós profissionais um sentimento e desrespeito e descanso.

**** *ind_1341 *estr_1 *sex_2 *ida_1 *prof_5

Exausta mentalmente e fisicamente.

**** *ind_1344 *estr_2 *sex_2 *ida_1 *prof_5

Preocupada.

**** *ind_1346 *estr_2 *sex_2 *ida_1 *prof_3

Tenho tido crise_de_ansiedade muitos. Mais frequentemente do que antes da pandemia.

**** *ind_1348 *estr_2 *sex_2 *ida_1 *prof_3

Foi necessário ajuste medicamentoso com antidepressivo dual e indutor do sono.

**** *ind_1349 *estr_2 *sex_2 *ida_1 *prof_14

A maioria das questões foram contempladas no estudo.

**** *ind_1352 *estr_1 *sex_2 *ida_2 *prof_14

Me sinto segura ao cuidado com o paciente, mas fico insegura com minha família, mantendo me distanciar devido_a suposta contaminação, porém fico triste, me sinto isolada.

**** *ind_1353 *estr_1 *sex_2 *ida_2 *prof_3

Medo, da reinfecção.

**** *ind_1354 *estr_1 *sex_2 *ida_2 *prof_3

Que as pessoas continuassem a ter calma e usar máscaras até acabar essa pandemia e questão de tempo.

**** *ind_1356 *estr_2 *sex_2 *ida_2 *prof_3

Meu nome é Janaína e vou relatar o que aconteceu na minha_casa nessa pandemia, marido é um sobrevivente da covid, ficou 64 dias, internado, sendo 42 no cti e hoje cuidado das sequelas que a covid deixou úlceras e fisioterapia junto com o home_care. Já realizei 2 exames negativados e não apresentei nenhum sintoma até a presente data, tento me resguardar o máximo para não me contaminar. Passei por momentos angustiante com a internação do meu marido e vi vários, sendo vencido pelo covid, não desejo a ninguém o que vivi durante essa pandemia.

**** *ind_1358 *estr_2 *sex_1 *ida_1 *prof_14

Estou bem.

**** *ind_1359 *estr_1 *sex_2 *ida_3 *prof_3

Tento me proteger psicológicamente das situações de estresse extremo pelas quais passei no período em que estive na triagem_da_emergência, por questões óbvias, para não sucumbir.

**** *ind_1362 *estr_2 *sex_2 *ida_1 *prof_14

Chateada.

**** *ind_1363 *estr_1 *sex_2 *ida_2 *prof_14

Eu sinceramente, me sinto privilegiada em poder contribuir na assistência aos pacientes internados com covid ajudando na sua recuperação através dos medicamentos administrados prescritos e também poder conversar com os mesmos, passando positividade e confiança para eles.

**** *ind_1364 *estr_1 *sex_2 *ida_2 *prof_7

Atualmente vejo profissionais_de_saúde sem epi, como o uso_ de_máscaras no ambiente_hospitalar com justificativas de ser imune por ter positivo, causando dúvidas aos pacientes em relação ao uso_de_máscaras.

**** *ind_1366 *estr_1 *sex_2 *ida_2 *prof_3

Me sinto insegura e às vezes assustada, principalmente com a questão_política que se encontra o país que os governantes roubam durante essa pandemia. Não incentivam a educação e a ciência.

**** *ind_1367 *estr_1 *sex_2 *ida_2 *prof_3

Neste momento de pandemia me sinto abençoada por deus e grata, pois durante todo o período_da_pandemia trabalho, coordenei uma equipe_de_trabalho nos fortalecendo uns aos outros em cada plantão, mesmo tendo a perda de um membro_da_equipe que foi impactante e confiantes na recuperação dos que foram acometidos pela doença e se recuperaram. Receosa pelos meus filhos e meu marido que faz parte do grupo_de_risco. As vezes muito entristecida pelo número_de mortes e sofrida pela perda de pessoas conhecidas. Confiante nas medidas de precaução no trabalho e no domicílio e que isso e uma fase e vai passar.

**** *ind_1368 *estr_2 *sex_2 *ida_1 *prof_3

Como gestora eu me sinto muito estressada e sobrecarregada. Apesar da minha atuação com o paciente covid ser pequena e controlada, sou responsável por um grande número_de trabalhadores e seus processos_de_trabalho. As demandas vêm por todos os lados e muitas vezes acho que não darei conta. Quando chega sexta feira, minha cabeça não aguenta mais e até pensar dói.

**** *ind_1371 *estr_1 *sex_2 *ida_1 *prof_3

Após o adoecimento engravidei e por isso, me considero em grupo diferenciado pois, já possuo anticorpos, no entanto, a falta de certeza de possível reinfecção e os males que poderia ou não trazer ao meu bebê ainda não são definidos.

**** *ind_1372 *estr_1 *sex_1 *ida_3 *prof_7

Estou triste em ver nosso país sem um ministro_da_saúde competente, alguém com formação vem saúde_pública. Não temos medidas concretas e uniformes. Estou triste por ter perdido vários colegas de turma. Faço minha parte tentando ajudar, superando as dificuldades e com cautela. O ritual imposto pela pandemia incomoda em demasia. Além do ritual para atendimento no consultório e ambulatório, ao chegar em_casa tenho que deixar os sapatos ensacados, colocar as roupas usadas direto no tanque junto a as usadas no consultório, lavar sem deixar que ninguém faça isso ou fique perto. Lavo tudo, coloco para secar. Depois disso vou para o banho então descarto a máscara após essa manipulação. Perde-se tempo, para impedir a contaminação possível.

**** *ind_1379 *estr_1 *sex_2 *ida_2 *prof_3

Mais segura.

**** *ind_1382 *estr_1 *sex_2 *ida_1 *prof_3

A população deveria ser melhor orientada pelos governantes.

**** *ind_1384 *estr_2 *sex_2 *ida_1 *prof_14

Solitária.

**** *ind_1388 *estr_1 *sex_2 *ida_1 *prof_7

Irritada com a falta_de_compromisso das pessoas, em_geral.

**** *ind_1393 *estr_2 *sex_2 *ida_2 *prof_14

Insegura, pois a assistência médica é incerta, cada médico ou instituição trata como acha que deve, não há um protocolo a ser seguido, de acordo com os estágios_da_doença, os remédios são difíceis e os testes mais ainda.

**** *ind_1403 *estr_2 *sex_2 *ida_2 *prof_3

Me sinto presa, e impossibilitada de executar planos esperados para 2020.

**** *ind_1408 *estr_2 *sex_2 *ida_1 *prof_11

Ansiosa.

**** *ind_1409 *estr_1 *sex_2 *ida_2 *prof_2

Me sinto sobrecarregada com acúmulo do trabalho e tarefas domésticas e cuidado de filho que está online.

**** *ind_1412 *estr_1 *sex_2 *ida_1 *prof_7

Um pouco triste porque a população em_geral está agindo como se não tivesse mais a circulação do vírus e consequentemente não estão com as precauções necessárias.

**** *ind_1413 *estr_2 *sex_2 *ida_2 *prof_1

Sentimentos de paralisação da vida, solidão, falta de perspectiva_de_futuro, vazio, frustração e incapacidade de voltar_às_atividades. Depressão maior crônica já diagnosticada. Obrigada.

**** *ind_1414 *estr_1 *sex_2 *ida_2 *prof_7

Eu continuei trabalho em parte dos empregos. Fui afastada do emprego_público.

**** *ind_1415 *estr_1 *sex_2 *ida_3 *prof_3

Estou bem, seguindo a vida com fé e mantendo as normas de segurança. Os vírus vem e vão.

**** *ind_1417 *estr_2 *sex_2 *ida_1 *prof_3

Me sinto desesperançosa pelo poder_público. Não somos valorizados, temos uma carga horária exaustiva e baixos_salários. Cuidamos de vidas, de pessoas que são importantes para alguém. A Enfermagem está zelando pela vida em todas as fases da vida. E infelizmente não somos valorizados, muitos de nós se foram, exercendo está profissão tão sublime. E para os governantes, não somos nada.

**** *ind_1419 *estr_2 *sex_2 *ida_1 *prof_11

O meu último mês foi um dos melhores por conta da flexibilização da quarentena apenas. Ela me permitiu fazer um esporte ao ar_livre na praia e isso fez com que o meu nível_de_estresse e ansiedade diminuísse consideravelmente.
